# Reconstructing the neuromuscular ground pattern of phylactolaemate bryozoans: new data from the Lophopodidae

**DOI:** 10.1186/s12862-022-02076-9

**Published:** 2022-10-19

**Authors:** J. Bibermair, T. S. Wood, R. Chaichana, T. Schwaha

**Affiliations:** 1 Department of Evolutionary Biology, University of Vienna, Vienna, Austria; 2 Department of Biological Sciences, Wright State University, Ohio, USA; 3 Department of Environmental Technology and Management, Faculty of Environment, Kasetsart University, Bangkok, Thailand

**Keywords:** Myoanatomy, Neuroanatomy, Lophophorata, *Lophopus*, *Lophopodella*, *Asajirella*, 3D reconstruction

## Abstract

**Background:**

The solely freshwater inhabiting Phylactolaemata is a sister taxon to all other bryozoans. Among phylactolaemates, Lophopodidae represents an early branching clade that is therefore crucial for ground pattern reconstruction. While more recent morphological data of most phylactolaemate families are present, data of lophopodids are scarce. The genus *Asajirella* especially, which was previously assigned to the family Pectinatellidae, lacks any detailed analysis with more recent morphological methods.

**Results:**

This study provides the first morphological analyses of three lophopodid species using serial-sectioning histology and 3D reconstruction, but also immunocytochemical stainings and confocal laserscanning microscopy. There are several lophopodid-specific traits in the nervous system such as the large ganglion with extensive lumen and two prominent protrusions referred to as epistomial horns. The epistome in all lophopodids is rather small and dome-shaped. Contrary to previous reports, we can confirm that duplicature bands insert at the tentacle sheath rather than the diaphragmatic sphincter in all phylactolaemates. The morphology of the digestive tract of lophopodids is identical to other phylactolaemates and possesses exclusively circular muscles.

**Conclusions:**

Altogether, this study fills significant gaps in our knowledge on phylactolaemate neuromuscular systems and general morphology. It shows that the insertion of the duplicature bands at the tentacle sheath and the circular musculature of the digestive tract to be the ground pattern in phylactolaemates. In addition, we found apomorphic characters for lophopodids such as the dome-shaped epistome with its musculature and the voluminous ganglion with its epistomial horns, which aid in defining and delineating the family.

## Background

Bryozoans are aquatic, colonial suspension-feeding lophotrochozoans. Phylactolaemata represents the sister taxon to all other remaining bryozoans, the Myolaemata [1–4]. The small group of phylactolaemates occur exclusively in freshwater habitats and have unmineralized skeletons. Recent molecular analyses support the notion of Phylactolaemata as sister-taxon to Myolaemata, which renders them essential for ground pattern reconstruction of the entire phylum [2, 3].

In general, bryozoan colonies are made up of individual zooids, which consist of a protective cystid and a retractable polypide. The former constitutes the body wall and comprises an inner, peritoneal layer and an outer epidermal layer that forms an acellular ectocyst, which in the case of phylactolaemates is gelatinous or chitinous/ encrusted [5]. In addition, muscle cells form a regular grid of body wall musculature that is embedded in the extracellular matrices of both cellular layers of the cystid. The retractable part of the zooid, the polypide, features a ciliated tentacle crown (lophophore) which is generally horse-shoe shaped in phylactolaemates and circular in myolaemates. At the lophophoral base, the mouth opening continues into a U-shaped digestive tract, which terminates outside of the lophophore. The digestive tract comprises the pharynx/oesophagus, cardia, caecum, pylorus and intestine. A funiculus and a retractor muscle that allows the polypide to retract into the cystid also connect the polypide to the cystid [6]. Protrusion is effectuated by increasing the coelomic pressure of the zooid via the body wall musculature. Phylactolaemates possess additional features such as the epistome: a ciliary, often flap-like protrusion above the mouth opening. Further, phylactolaemates produce internal buds, called statoblasts that are crucial for taxonomic purposes [7]. In contrast to myolaemates, phylactolaemates are a small group of only approx. 80 extant species subdivided into 6(-7) families [8]. Traditionally, two colony morphologies are recognised: Families like Lophopodidae, Cristatellidae and Pectinatellidae form colonies with clustered zooids that possess a hyaline/gelatinous ectocyst. In contrast, Plumatellidae and Fredericellidae show a serial arrangement of the zooids and feature chitinous/ encrusted cystids [7, 9]. The early branching Stephanelidae represent an intermediate from, as they have serial arranged zooids but have a jelly-like ectocyst [10]. Gelatinous families were traditionally considered derived whereas recent studies shows that Lophopodidae branch off early and cluster together with stephanellids [11, 12]. Thus, Lophopodidae are crucial for reconstructing the ancestral character state of phylactolaemates. Although recent morphological data, including the neuromuscular system, are available for most families (Cristatellidae: [13–18]; Fredericellidae, Plumatellidae: [18–22]; Pectinatellidae: [14], Stephanellidae: [10, 23]), recent studies of Lophopodidae are scarce (e.g. [24, 25]). Lophopodids comprise three different genera: *Lophopus* (*Lophopus crystallinus* e.g., [24, 26, 27]). The genus forms in small fan-shaped colonies, which include tapered/spindle-shaped statoblasts [9]. *Lophopodella* comprises several species [28], but only *Lophopodella carteri* has been studied in detail [29, 30]. Compared to *Lophopus*, *Lophopodella* colonies are circular and grow smaller. All species of the latter feature a varying number of spines on their statoblast [9, 31]. The third genus, *Asajirella*, is monotypic and was first erected in the late twentieth century [32]. The species, *Asajirella gelatinosa*, was originally assigned to the genus *Pectinatella* [33], based on saddle shaped statoblasts and compound colonies. The more recent assignment to lophopodids is based on histochemical staining properties, similar karyotypes, and also similar colony, larva, and statoblast morphology that unite these three genera [32]. However, owing to the early-branching position of the family the lack of newer morphological data on zooidal level hamper ground pattern reconstruction. Moreover, lophopodids are the last family where details of the neuromuscular system remain unknown, and certain character states such as the gigantic ganglion and lumen or the vestigial epistome observed in *Lophopus* [24] require a more profound analysis of its members. Particularly data on *A. gelatinosa* are very patchy [33–37]. Ultimately, this study complements the available morphological data by analysing *A. gelatinosa* with immunocytochemical stainings and confocal microscopy, but also serial sectioning and 3D-reconstruction techniques. The new data allow more insight into the lophopodid and thus phylactolaemate ground pattern. Additional new data of a member of the genus *Lophopodella* are also included in this study to further assess the morphological ground pattern for this family.

## Methods

Colony pieces of *A. gelatinosa* were collected from Phetchaburi province and ponds from the Sakhon Nakon area, both in Thailand, in February 2020. Before fixation, the samples were photographed using a Nikon Z6 digital camera (Nikon, Tokyo, Japan) mounted on Wild M420 stereomicroscope. For confocal microscopy, colony pieces of *A. gelatinosa* were fixed in 4% paraformaldehyde in 0.1 M phosphate buffer (PB) whereas for light microscopy and histology, samples were fixed in 2,5% glutaraldehyde in 0.1 M PB (both pH 7.3). Until further processing, samples were stored at 4 °C in 0,1 M PB with 0.1% sodium azide (NaN_3_).

Several colonies of *Lophopodella carteri* were collected in Dayton, Ohio (USA) and fixed according to methods described above. Samples of *Lophopus crystallinus* were only available as serial-sections and not for fluorescence stainings.

### Immunocytochemical staining and confocal microscopy

To increase tissue permeability, small colony pieces of *A. gelatinosa* were separated from their gelatinous ectocyst and further dissected into single zooids. In addition, vibratome sections of 80–100 µm thickness of undissected colony pieces were produced using a Leica, VT 1200 S vibratome (Leica Microsystems, Wetzlar, Germany). Vibratome sections and dissected zooids were permeabilized overnight in PB including 2% Triton-X 100 and 2% dimethylsulfoxide (PBT). For tubulin staining, specimens were incubated in primary antibodies against acetylated α-tubulin (Cat# T6793, Sigma-Aldrich, St. Luis, MO, USA) at a dilution of 1:800 in PBT for 24 h. Subsequently, secondary antibodies (Alexa Flour 568 anti-goat, Cat# A11004, Thermo Fisher Scientific) were applied at a dilution 1:300 in PBT for 4–24 h. In addition, specimens were simultaneously stained for f-actin using Alexa flour 488 phalloidin (dilution 1:40; Cat# A12379, Thermo Fisher Scientific) and nuclear-counterstained with 4’,6-diamidino-2-phenylindole (DAPI) (Invitrogen, Carlsbad, CA, USA). Between each step, samples were rinsed several times in PBT respectively PB in the last rinse. Samples were mounted on standard object slides and coverslips using Flouromount G (Southern Biotech, Birmingham, AL, USA) as mounting medium for whole mounts and Mowiol (Merck, 475904) for vibratome sections.

Samples were scanned with a Leica TCS SP5 II confocal laser scanning microscope (Leica Microsystems, Wetzlar, Germany). Image stacks were further analysed with the software Amira 6.3 and 6.4 (Thermo Fischer Scientific). Maximum intensity projections were produced using the image orthoprojection module of Amira and Fiji [38]. Volume renderings were produced using the VolumeRendering module of Amira 6.3 and 6.4.

### Histology

Prior to embedding, stored samples were postfixed in 1% aqueous osmium tetroxide (OsO_4_) for 1 h and rinsed at least 3 times in double distilled water (20 min each). Osmification was followed by dehydration with acidified 2,2-Dimethoxypropane (DMP, 30 min) and rinsing in pure acetone at least 3 times (20 min each). Dehydrated specimens were then infiltrated with low viscosity resin (LVR, Agar Scientific, Stansted, Essex, UK), using acetone as intermediate (overnight). Finally, the infiltrated samples were embedded in silicone moulds. Polymerisation of the resin blocks was done at 60 °C for 12 h.

For histological investigations, ribbons of serial sections (1 µm) were produced, using a Histo Jumbo knife (Diatome, Nidau, Switzerland) mounted on a Leica UC6 ultramicrotome (Leica Microsystems, Wetzlar, Germany) (see [39]). Serial sections were stretched on the object slide at 70 °C and stained with toluidine blue (1%, 40 s, 60 °C). The semi-thin sections were analysed and photographed using a Nikon Ni-U compound microscope (Nikon, Tokyo, Japan) with a mounted Nikon Ds-Ri2 camera.

### 3D-Reconstruction and image processing

Images unsuitable for reconstruction were replaced by neighbouring ones using the ‘stack sorter’-tool of FIJI [38]. The image stack was then converted into grey scales with FIJI. Next, the processed image stack was imported into the Amira 3D reconstruction software, where it was semi-automatically aligned using the AlignSlices tool. Polypides and regions of interest were segmented manually in the segmentation editor with interpolations between the sections. The SurfaceGen module of Amira was used to create surfaces, which were then optimised via iterative triangle reduction combined with smoothing steps. Snapshots were exported from Amira and further processed using photoshop and FIJI.

## Results

### Asajirella gelatinosa

#### General morphology

**Fig. 1:**
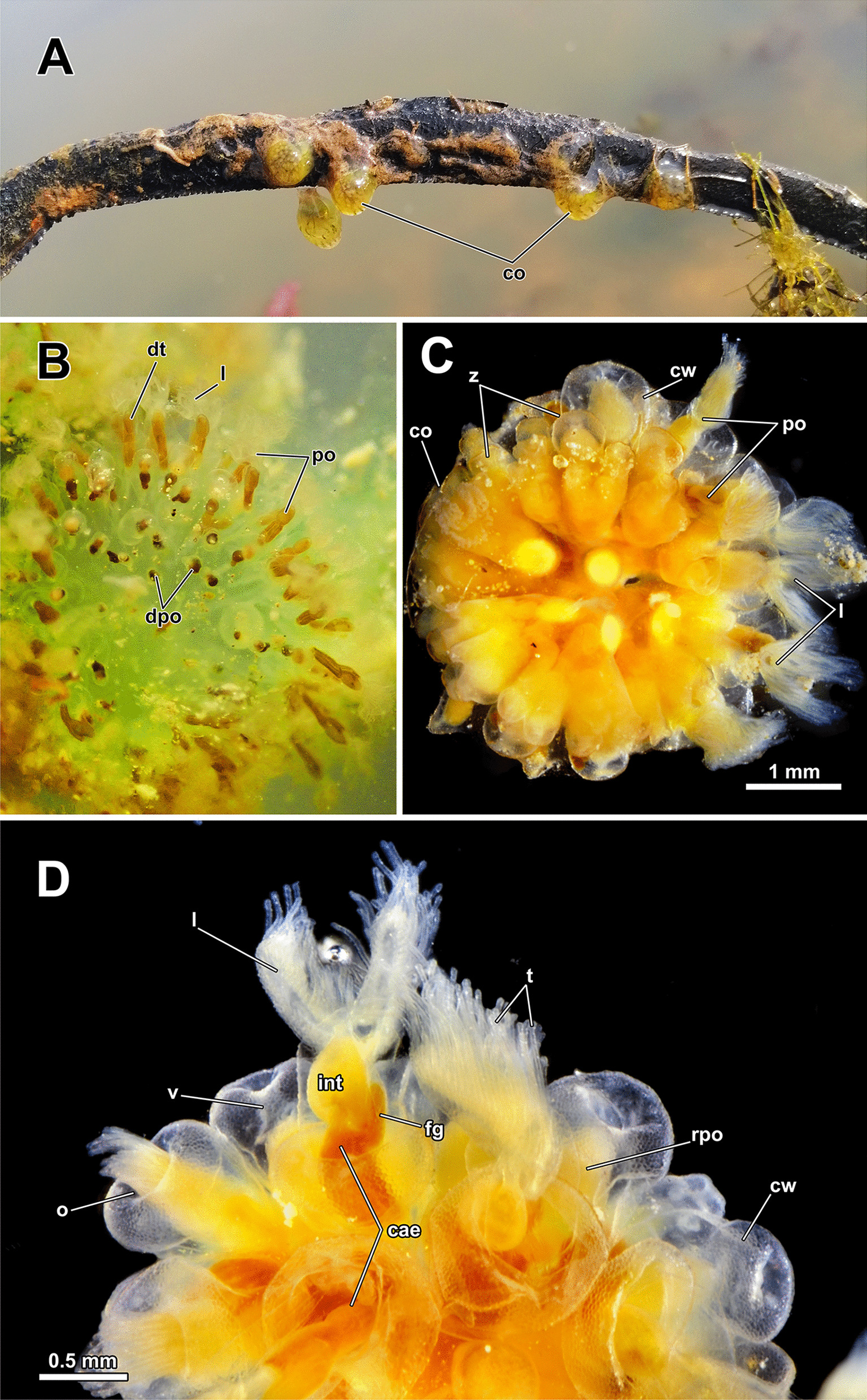
General appearance of *Asajirella gelatinosa*. **A** Several small colonies growing on natural substrate such as wood. **B**, **C** Individual colonies, with degenerated polypides in the center and active polypides arranged in the periphery. The digestive tract is always straight. **D** Detail of several zooids, with a large protruded lophophore and all components of the digestive tract differentiable. *cae* caecum, *co* colony, *cw* cystid wall, *dpo* degenerated polypide, *dt* digestive tract, *fg* fore gut, *int* intestine, *l* lophophore, *o* orifice, *po* polypide, *rpo* retracted, polypide, *t* tentacle, *v* vestibulum, *z* zooid

Colonies of *A. gelatinosa* were found growing on wood (Fig. 1A) and consist of clustered circularly arranged autozooids (Fig. 1B–D). Colonies show a high density of individual zooids. Functional zooids are mostly situated in the periphery of the colony, and small protrusions closer to the center represent degenerated zooids (Fig. 1B, C). Distally, the zooids possess a transparent epidermis with a sometimes mamillated appearance owing to epidermal gland cells (Fig. 1D).

#### Body wall/cystid

**Fig. 2:**
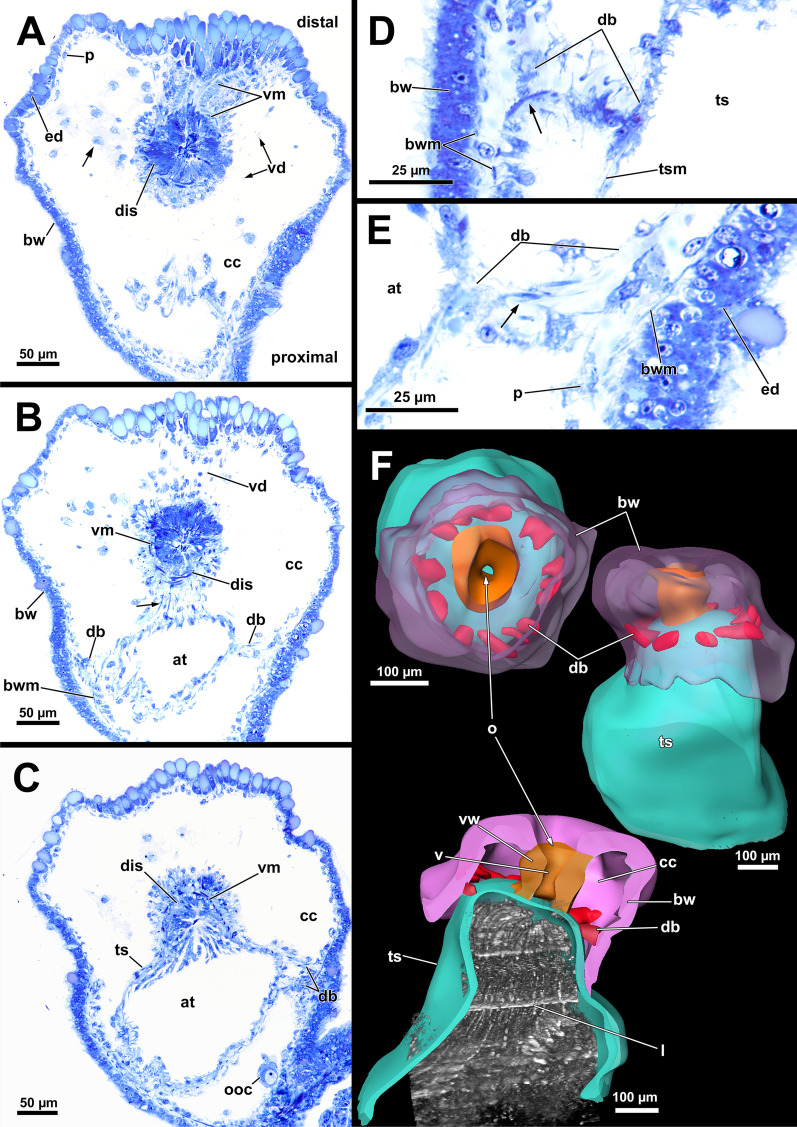
Histological details and 3D-reconstruction of the distal area of *Asajirella gelatinosa*. **A**–**C** Oblique sections showing the vestibulum as invagination with circular muscles adjacent to the diaphragmatic sphincter muscle (**A**). The latter separates the vestibulum from the atrium (**B**) and continues into the tentacle sheath (**C**). Vestibulum dilatators are present in the distal area and connect the body wall to the vestibulum. In addition, numerous coelomocytes are present (**A**, arrow). **D**, **E** Details of the duplicature bands show the longitudinal muscles of the body wall project into the tentacle sheath (arrow) via the duplicature band connected to the tentacle sheath. **F** 3D-reconstruction of the apertural area shows the general anatomy of the latter. the vestibular wall is followed by the tentacle sheath. The duplicature bands are arranged in a circular plain around the tentacle sheath. *at* atrium, *bw* body wall, *bwm* body wall musculature, *cc* coelomic cavity, *db* duplicature bands, *dis* diaphragmatic sphincter, *ed* epidermis, *l* lophophore, *o* orifice, *ooc* oocytes, *p* peritoneum, *ts* tentacle sheath, *tsm* tentacle sheath musculature, *vd* vestibulum dilatators, *v* vestibulum, *vm* vestibular muscles, *vw* vestibular wall

**Fig. 3:**
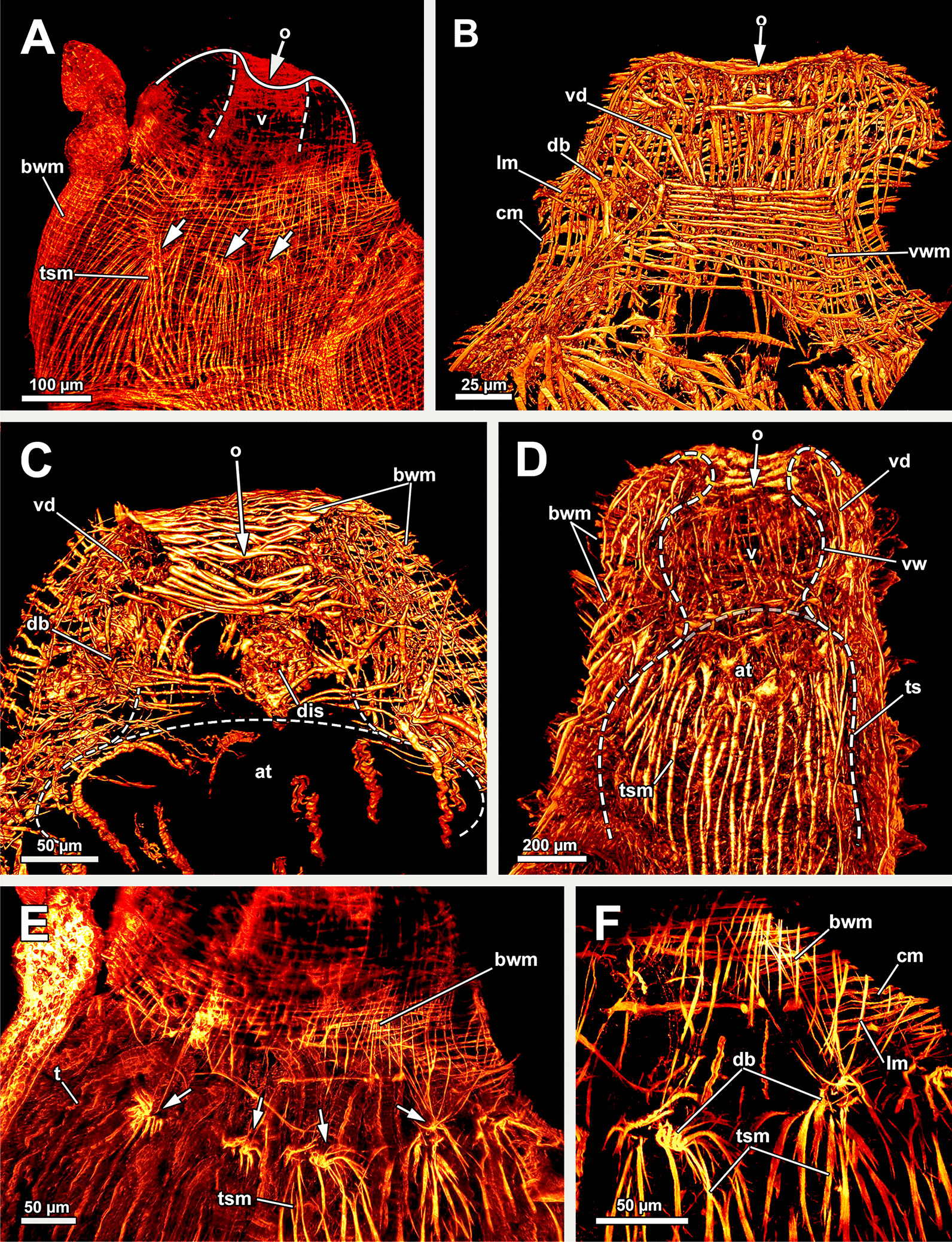
Musculature of the apertural area of *Asajirella gelatinosa* stained for f-actin, visualised by volume renderings and ortho projections. **A** Overview scan of the distal body wall shows the orthogonal body wall musculature. Arching over the tentacle sheath are several duplicature bands (arrows). **B**–**D** longitudinal vibratome sections of the apertural area. The muscles of the vestibular wall consist primarily of circular muscles (**B**) that are also very densely arranged around the orifice (**C**). Vestibulum dilatators were detected in the distal area of the coelomic cavity. The vestibular wall is proximally followed by the tentacle sheath, which shows thick longitudinal muscles (**D**). **E**–**F** Details of the duplicature bands. Prominent longitudinal muscles of the tentacle sheath and comparatively slender muscles of the body wall join each other in the duplicature bands (arrows), that are arranged at the same level between body wall and tentacle sheath. *bwm* body wall musculature, *cm* circular muscles, *db* duplicature bands, *dis* diaphragmatic sphincter, *lm* longitudinal muscles, *o* orifice, *t* tentacle, *ts* tentacle sheath, *tsm* tentacle sheath musculature, *vd* vestibulum dilatators, *v* vestibulum, *vw* vestibular wall, *vw*m vestibulum muscles

The cystid includes a regular grid of body wall musculature between the epidermis and the peritoneum (Fig. 2A–E). The body wall musculature is exclusively bilayered and comprises an outer layer of circular and an inner layer of longitudinal muscles (Fig. 3A, B, E, F). At the distal end of the cystid, the body wall including its musculature continues into the apertual area (Fig. 3B, C).

#### Lophophore and digestive tract

**Fig. 4:**
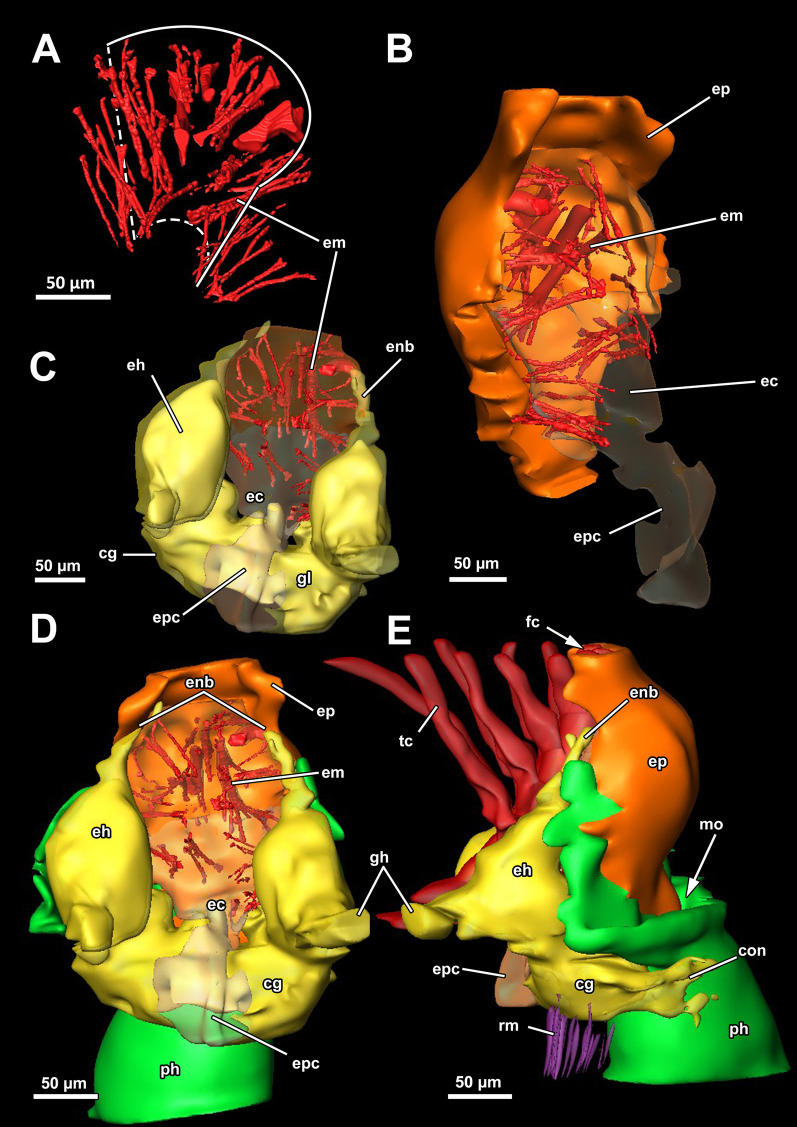
Section-based 3D-reconstruction of the lophophoral base of *Asajirella gelatinosa*. **A** Anal view of the transverse epistome muscles with schematic outline of the epistome. **B** Oblique anal view of the epistome shows the epistome coelom ascending from a narrow canal between the gut shanks. **C** Anal view of the cerebral ganglion with prominent epistomial horns bilaterally ascending into the epistome. Transverse epistome muscles project in between the epistomial horns. **D** Anal view of the lophophoral base showing the narrow canal of the epistome coelom above the cerebral ganglion. The epistomial horns continue distally as epistomial neurite bundles. **E** Lateral view of the lophophoral base showing the dome-like appearance of the epistome and the tentacles supplied by the forked canal above the epistome in the lophophoral concavity. Ganglionic horns project anally of the cerebral ganglion, a retractor muscle inserts proximally of the latter. *cg* cerebral ganglion, *con* circumoral nerve ring, *ec* epistome coelom, *eh* epistomial horns, *em* epistome musculature, *enb* epistomial neurite bundles, *ep* epistome, *epc* epistome canal, *fc* forked canal, *gl* ganglion lumen, *gh* ganglionic horns, *ph* pharynx, *rm* retractor muscle, *tc* tentacle coelom

**Fig. 5:**
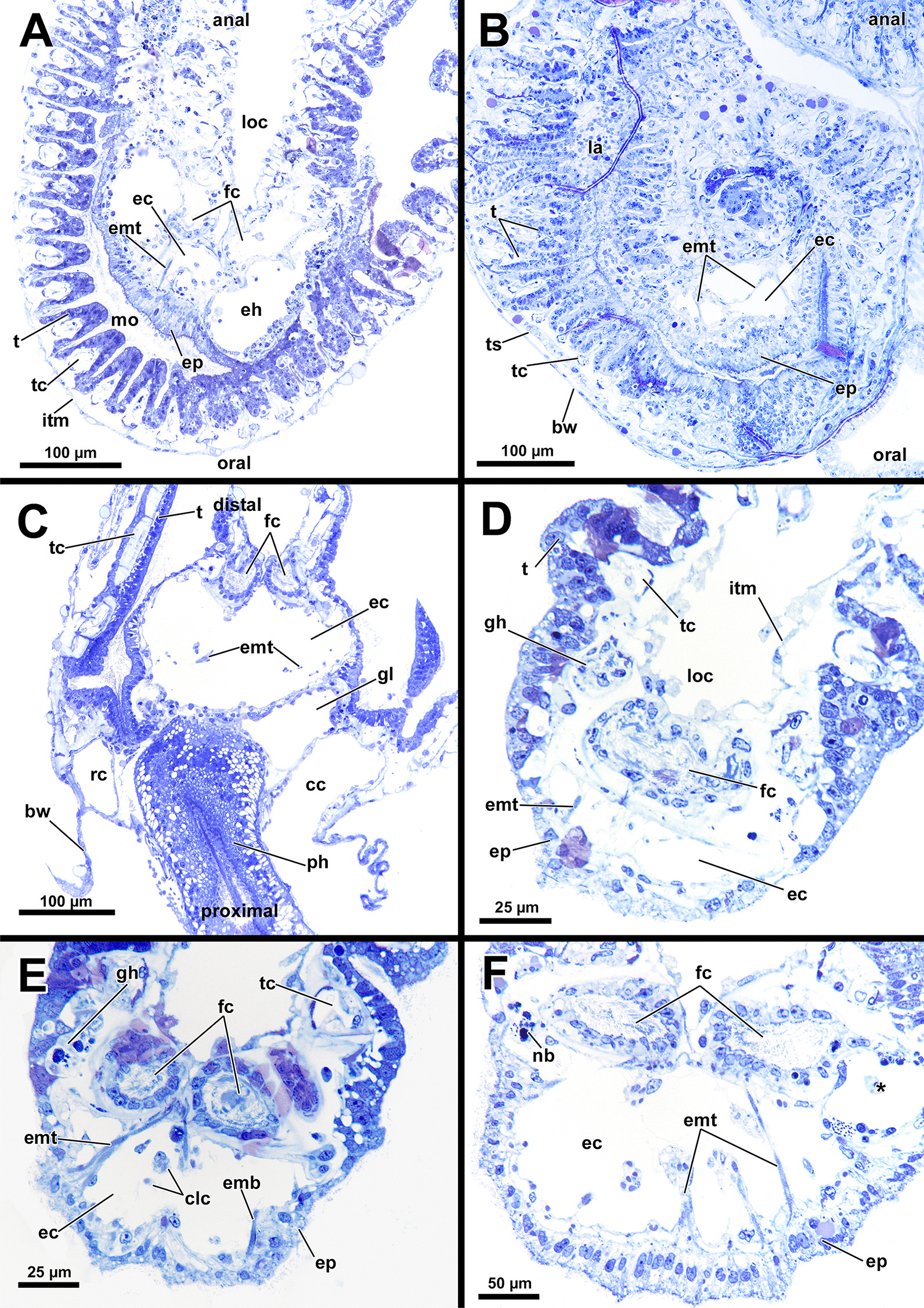
Histological details of the lophophoral base of *Asajirella gelatinosa*. **A**, **B** Cross section through the lophophore base above the cerebral ganglion of a protruded (**A**) and retracted (**B**) zooid. The epistomial horns bear a large lumen. The epistome coelom ascends via a canal between the epistomial horns. **C** Longitudinal section through the lophophore base of a protruded zooid. Centrally, the epistome coelom extends to a large cavity. Proximally the latter is flanked by projections of the cerebral ganglion and the ring canal, whereas the ciliated forked canal ascends distally. **D**–**E** Detailed cross sections through the epistome and lophophoral concavity at different heights. Distally, the coelom (forked canal) of three tentacles in the lophophoral concavity are connected (**D**), whereas they are separate more proximally (**E**, **F**). In addition, the epistome includes a muscle basket in its lining and muscles traversing the epistome coelom. *bw* body wall, *cc* coelomic cavity, *clc* coelomocytes, *ec* epistome coelom *eh* epistomial horns, *emb* epistome muscle basket, *emt* traversing muscles of the epistome, *ep* epistome, *fc* forked canal, *gl* ganglion lumen, *gh* ganglionic horns, *itm* intertentacular membrane, *la* lophophoral arm, *loc* lophophoral concavity, *mo* mouth opening, *nb* neurite bundle, *ph* pharynx, *rc* ring canal, *t* tentacle, *tc* tentacle coelom, *ts* tentacle sheath, *asterisk* lumen of the epistomial horn

**Fig. 6:**
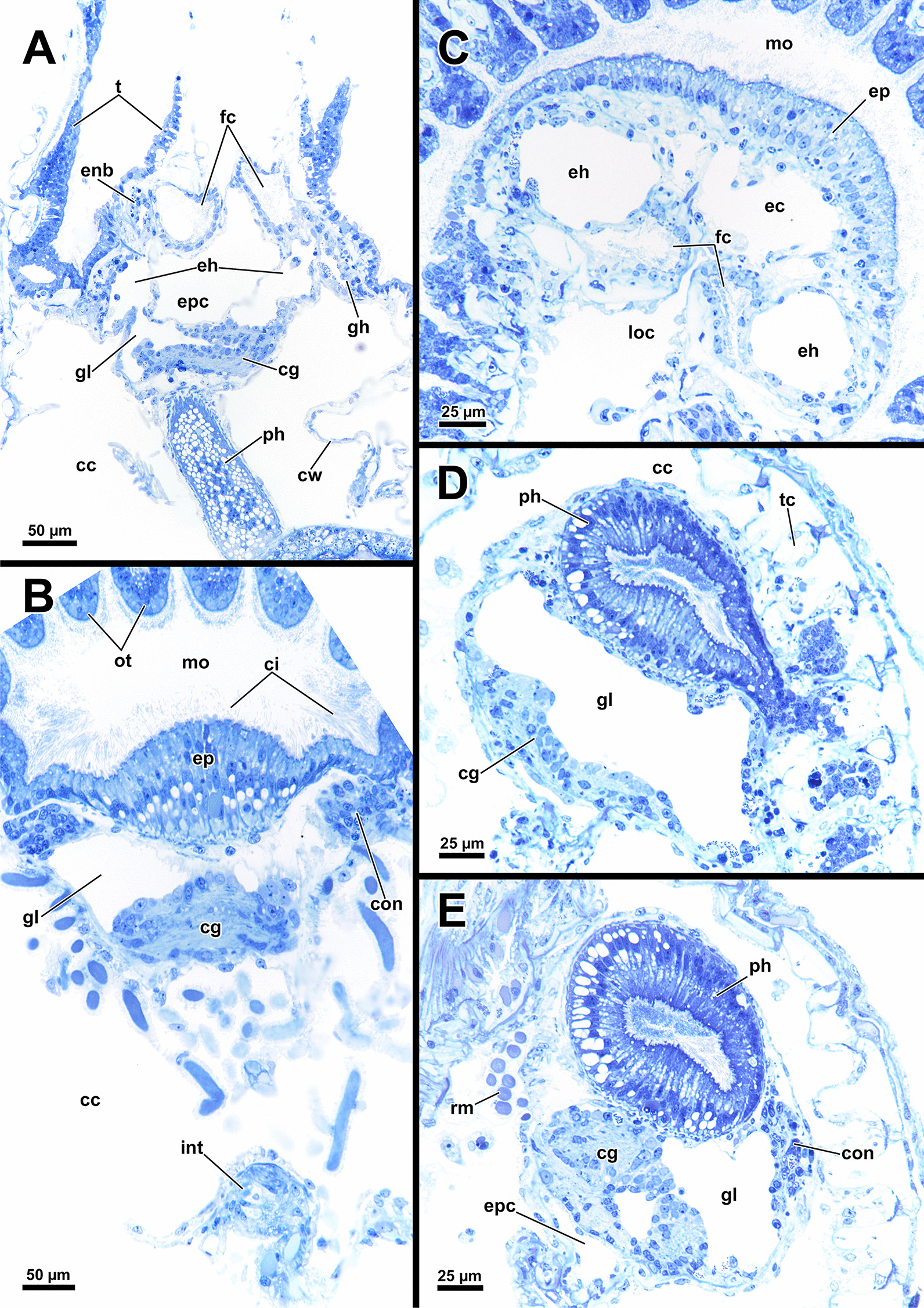
Histological details of the central nervous system of *Asajirella gelatinosa*. **A** Oral view of the lophophoral base. The cerebral ganglion is located at the anal side of the pharynx and shows several projections with the ganglionic horns projecting into the lophophoral arms and epistomial horns into the epistome. Above the epistome coelom, the ciliated forked canal is shown. **B** Cross-section through the lophophoral base shows the cerebral ganglion and the intestine. Several bundles of the retractor muscle insert at the peritoneal lining of the ganglion. **C** Cross section through the distal area of the lophophoral base including two epistomial horns with large lumina. **D** Cross section through the central area of the cerebral ganglion showing the large size of the ganglionic lumen. Neuronal tissue is located on the anal wall of the ganglion. **E** Cross-section through the proximal area of the cerebral ganglion showing dense neuronal tissue. The circumoral nerve ring projects orally around the pharynx. *cc* coelomic cavity, *cg* cerebral ganglion, *ci* cilia, *con* circumoral nerve ring, *cw* cystid wall, *ec* epistome coelom, *eh* epistomial horns, *enb* epistome neurite bundles, *ep* epistome, *epc* epistome canal, *fc* forked canal, *gl* ganglion lumen, *gh* ganglionic horns, *int* intestine, *loc* lophophoral concavity, *mo* mouth opening, *ot* oral tentacles, *ph* pharynx, *rm* retractor muscle, *t* tentacle, *tc* tentacle coelom

**Fig. 7:**
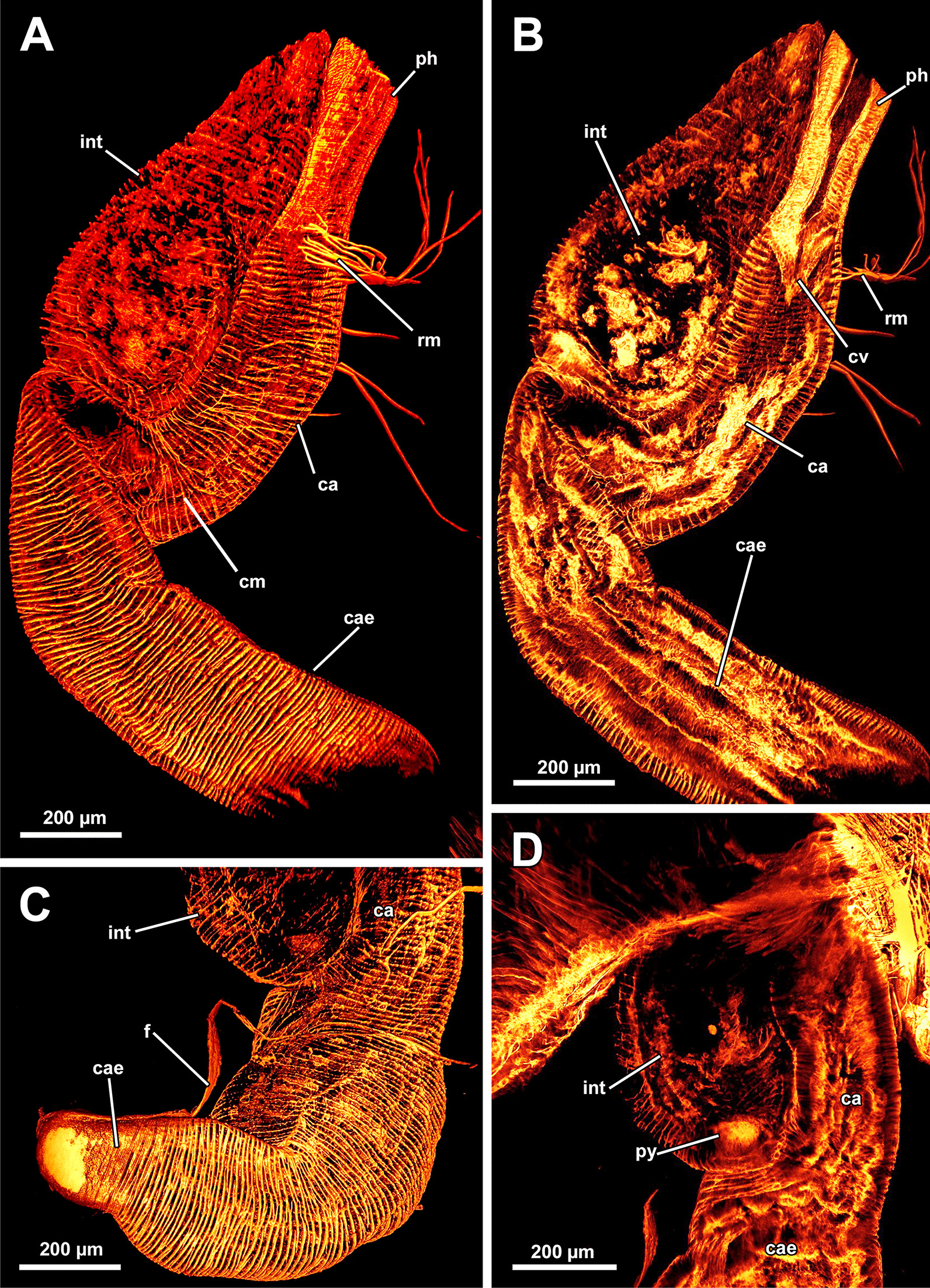
Musculature of the digestive system of *Asajirella gelatinosa* stained for f-actin, visualised by volume renderings and ortho projections. **A** Lateral view of the digestive tract shows the circular musculature of the digestive tract with different densities in different parts and several attachments of retractor muscles **B** Sagittal section of the digestive tract muscles. The densest arrangement of muscles is found in the pharynx, which continues in the cardia via the cardiac valve. **C** The proximal tip of the caecum shows a funiculus that stains positive for f-actin as well. **D** Section through the distal half of the digestive tract shows the ascending arm of the caecum entering the intestine via the narrow pylorus. *ca* cardia, *cae* caecum, *cm* circular musculature, *cv* cardiac valve, *f* funiculus, *int* intestine, *ph* pharynx, *py* pylorus, *rm* retractor muscle

*A. gelatinosa* has a large, horseshoe-shaped lophophore with a mean width of 0.5 mm (SD 0.058 mm) and a mean hight of 0.86 mm (SD 0.1 mm, distance from the lophophoral base to the tip of the tentacles). The mean length of the lophophoral arms is 0.71 mm (SD 0.044 mm) and in total the lophophore bears at least 75 tentacles (Fig. 1C, D). At the bottom of the lophophore lies the mouth opening. Above the latter, the epistome protrudes as a small bulge (Fig. 4E). The cerebral ganglion is located beneath the epistome, adjacent to the pharyngeal epithelium (Fig. 5C; Fig. 6A, B). The digestive tract has a foregut consisting of a pharynx and esophagus, which is separated from the cardia by a cardiac valve. Proximally, the gut continues via the cardia into the caecum and exits via a pyloric area into the intestine (Fig. 7). In retracted condition the midgut (cardia and caecum) remains unfolded in *A. gelatinosa*.

### Myoanatomy

#### Apertural and tentacle sheath musculature

**Fig. 8:**
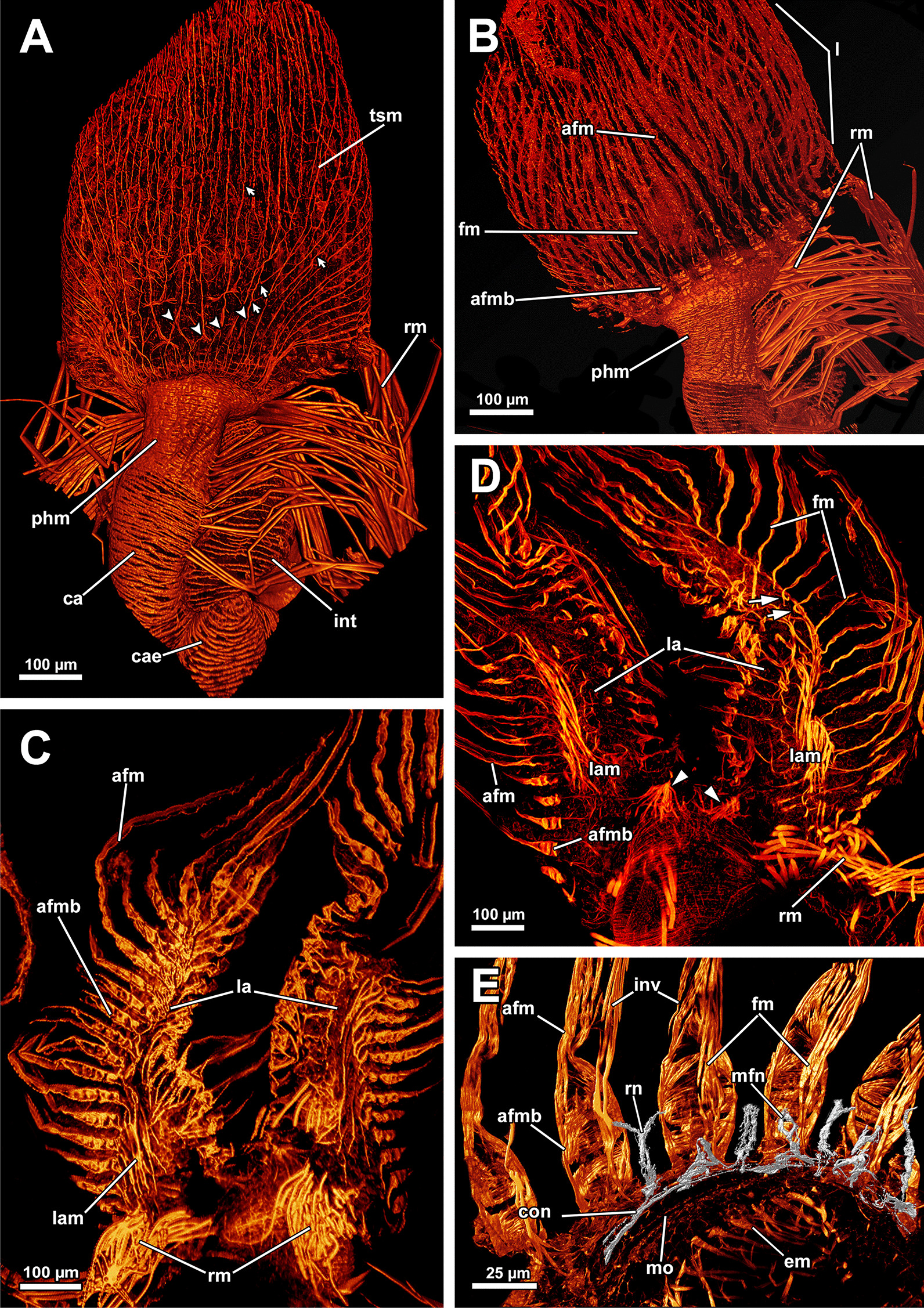
Myoanatomy of the lophophoral system of *Asajirella gelatinosa* stained for f-actin and against acetylated-α-tubulin, visualised as volume renderings. **A** Oral view of a polypide, retracted into the tentacle sheath. The tentacle sheath consists of longitudinal muscles with proximal bifurcations. The dense circular musculature of the foregut is followed by the less dense arrangement in the cardia. Several muscle bundles of the retractor muscle insert at various areas of the digestive tract. **B** Oral view of the lophophore showing prominent muscle bases of the oral tentacles and abfrontal muscles ascending into the tentacles. **C**, **D** Distal view on the lophophoral arms showing the prominent lophophore arm muscles becoming slender along the oral- anal axis as tentacle muscles branch off. At their origin a pair of retractor muscle inserts. Abfrontal (**C**) and frontal (**D**) muscles of the tentacle associated with the lophophore arm muscles. **E** Anal view of the oral tentacles showing the large bases of the abfrontal tentacle muscles including several median bands. In contrast to the former, the frontal muscles lack prominent bases. Both tentacle muscles ascent as inverted ‘v’ muscles. Interdigitating with the tentacle muscles, radial nerves branch off a circumoral nerve ring while mediofrontal nerves are located next to the frontal tentacle muscles. *afm* abfrontal tentacle muscle, *afmb* abfrontal tentacle muscle base, *ca* cardia, *cae* caecum, *con* circumoral nerve ring, *em* epistome musculature, *fm* frontal muscle, *int *intestine, *inv* inverted ‘v’ muscle, *l* lophophore, *la* lophophore arm, *lam* lophophoral arm musculature, *phm* pharynx musculature, *rm* retractor muscle, *rn* radial nerve, *tsm* tentacle sheath musculature

The orifice, through which the polypide is protruded, is located at the distalmost area of the zooids (Figs. 1D, 2A–C, F) and can be widened and narrowed via the regular body wall and apertural musculature. When the polypide is retracted, a vestibulum forms as invagination of the body wall that is limited by the vestibular wall. In *A. gelatinosa*, the vestibular wall muscles consist of prominent circular muscles and comparatively slender longitudinal muscles (Figs. 2A, 3B–D). When the polypide is retracted, the vestibular wall continues proximally into the tentacle sheath (Figs. 2A–C, 3C, D). The epidermal cells of the vestibular epithelium are rather voluminous and stain intensely on sections in *A. gelatinosa*. A diaphragmatic sphincter muscle is located at the junction of the vestibular epithelium to the tentacle sheath and shows circular to oblique muscle fibres (Figs. 2A–C, 3C). The musculature of the tentacle sheath consists predominantly of longitudinal muscles (Figs. 3D–F, 8A) that bifurcate towards the lophophoral base (Fig. 8A, arrowheads). Several additional muscle bundles branch off individually from the longitudinal ones and show a rather oblique traverse arrangement (Fig. 8A, arrows).

Additional apertural muscles are present as the vestibulum dilatators and the duplicature bands. The former are single muscle bundles connecting the distal body wall with the vestibular wall over the entire range of the vestibulum (Figs. 2A–C, 3B–D). In contrast to the dilatators, the duplicature bands are peritoneal bands, circularly arranged in a plane. They originate from the body wall and insert at the tentacle sheath (Fig. 2B–E, F). The longitudinal muscles of the body wall are continuous with those of the duplicature bands (Figs. 2 D, E, 3E, F).

#### Lophophore and digestive tract

**Fig. 9:**
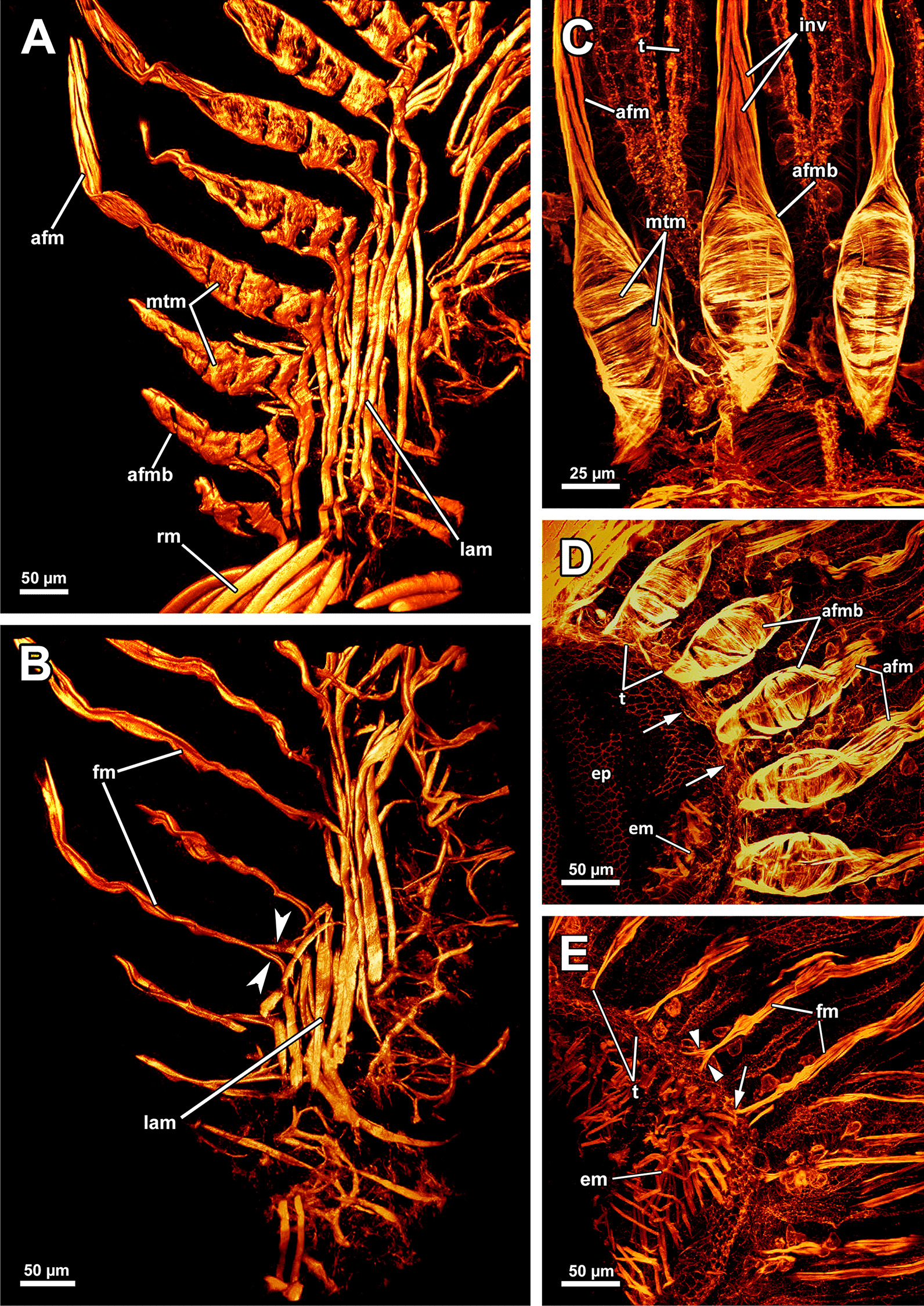
Details of the lophophore musculature of *Asajirella gelatinosa* stained for f-actin, visualised by volume renderings and ortho projections. **A**, **B** Detailed scan of the lophophoral arms showing prominent bases of the abfrontal tentacle muscles (**A**) associated to the lophophoral arm musculature. The bases of the abfrontal muscles include 4–5 median traversal bands. The frontal muscles lack a prominent base instead rise from two roots that are likewise connected to the lophophoral arm muscles (**B**). **C**–**E** Closeup view on lateral (**C**) and oral (**D**) tentacles showing the bases of the abfrontal muscles including several (4–5) distinct median bands. The frontal muscles of the oral tentacles show at least two rootlets and consist of slightly twisted muscle bundles as well. *afm* abfrontal tentacle muscle, *afmb* abfrontal tentacle muscle base, *em* epistome musculature, *ep* epistome, *fm* frontal tentacle muscle, *inv* inverted ‘v’ muscle, *lam* lophophoral arm musculature, *mtm* median transverse muscle, *rm* retractor muscles, *t* tentacle

**Fig. 10:**
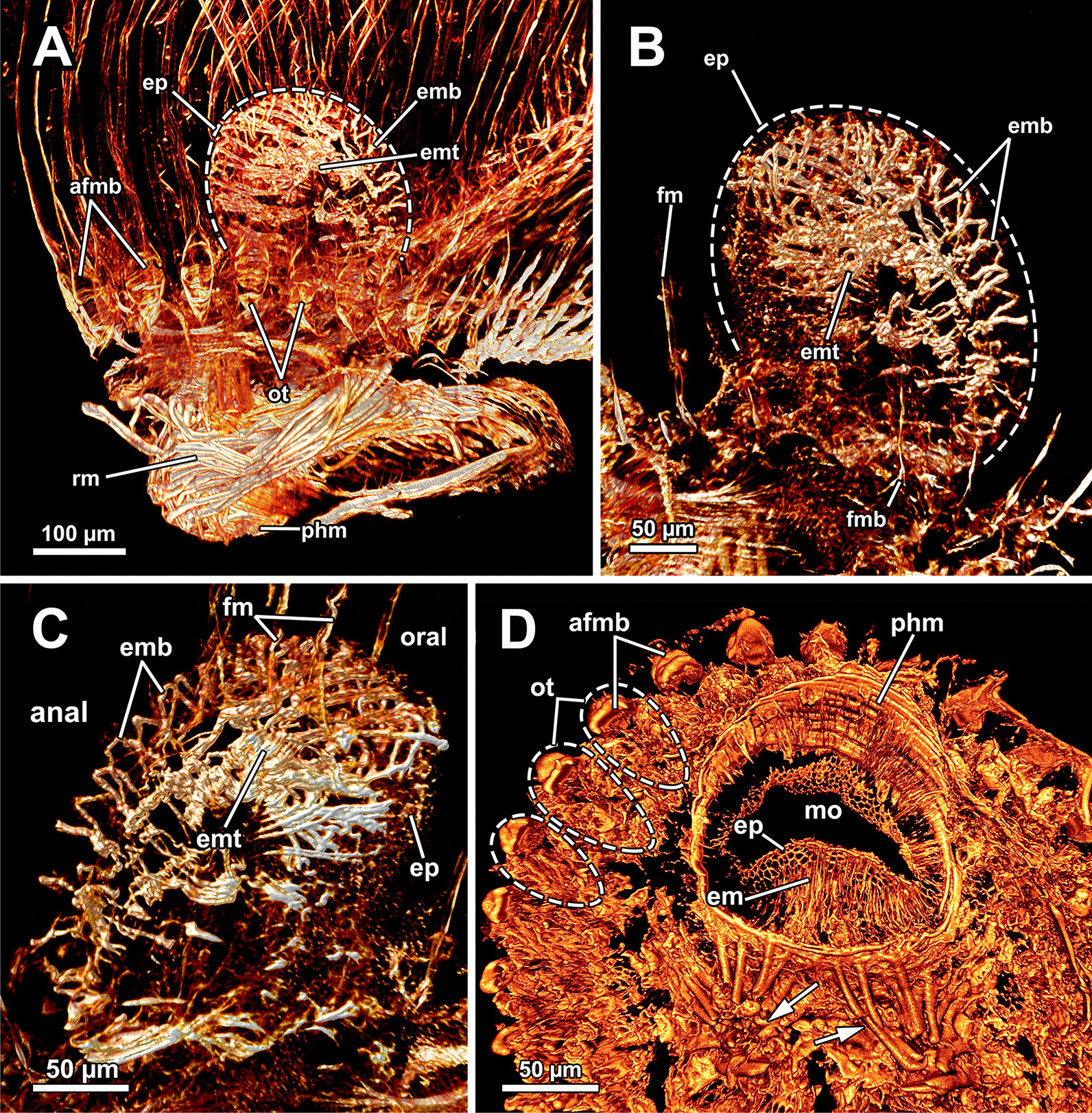
Musculature of the epistome of *Asajirella gelatinosa*, stained for f-actin. **A** Oral view of the lophophore base with the epistome muscles above the mouth opening. The circular muscles of the pharynx are displayed with the prominent retractor muscles in front. **B** Detailed oral view of the epistome with a peripheral muscular basket and central traverse muscle fibres. **C** Anal view of the epistome with the peripheral muscle basket of and comparatively thick traversing muscles in the center. Musculature of the tentacles in the lophophoral concavity are associated with traverse epistome muscles. **D** Vibratome section of the lophophoral base. The epistome appears as bulge at the anal side of the mouth. Thick muscle bundles project in the direction of the anus (arrows). *afmb* abfrontal tentacle muscle base, *em* epistome musculature, *emb* epistome muscle basket, *emt* traverse muscles of the epistome, *ep* epistome, *fm* frontal tentacle muscle, *fmb* frontal tentacle muscle base, *mo* mouth opening, *ot* oral tentacles, *phm* pharynx musculature, *rm* retractor muscle

The lophophoral base includes several muscle systems of which the lophophore arm muscles are the most prominent (Figs. 8C, D, 9 A, C). They originate from the lophophoral base and run anally into the distal part of the lophophore arms. Since *A. gelatinosa* has an unusually large lophophore, the respective musculature consists of numerous muscle bundles. The muscle fibres of the arm muscles decrease in number in distal direction (Fig. 9A, C). The outer, abfrontal tentacle muscles emanate from a large muscular base that includes several (≥ 5) transversal muscle bands in *A. gelatinosa* (Fig. 9C). The abfrontal muscle itself appears in the form of stacked inverted ‘V’s and thus referred to as `inverted V muscle` (Fig. 9B). This appearance is probably underlined by twisted muscle bundles ascending to the top of the tentacle (Fig. 9A). Besides the abfrontal muscle, each tentacle also possesses an inner, frontal muscle (Figs. 8C–E; 9C, E). In contrast to the abfrontal side, there are no prominent muscular bases on the frontal side. Instead, two rootlets comprise the base of the rather thin frontal tentacle muscles (Fig. 9E), which appear as inverted ‘V’ muscle as well (Fig. 8E). Regardless of their different dimensions, both tentacle muscle bases of *A. gelatinosa* are connected to the lophophore arm musculature (Fig. 9A, C). This configuration of tentacle muscles is found in the lateral tentacles and the lophophore arms tentacles. The few oral tentacles are similar but lack any connection to the lophophore arm muscles and are instead anchored to the distalmost pharynx epithelium (Fig. 9D, E). The tentacles situated in the lophophoral concavity differ in their frontal muscles and are connected to the musculature of the epistome (Fig. 10C). Additional lophophoral base muscles originate from the anal side of the pharynx (Fig. 10A) and traverse towards the intestine (Fig. 6B).

All regions of the gut include exclusively circular musculature (Fig. 7). The muscles are particularly dense in the pharynx and the proximal tip of the caecum and less dense in the intestine (Figs. 7, 8A, B). Multiple prominent retractor muscle fibres emanate from the body wall and attach at several locations of the digestive tract and the lophophoral base area (Fig. 8A, B), including the cerebral ganglion (Fig. 4E), and i.e. pharynx/esophagus, cardia, caecum (Figs. 7, 8A, B). Also, the funiculus, a peritoneal strand, inserts at the proximal end of the caecum (Fig. 7C) and connects the polypide to the cystid wall. Longitudinal muscles run along the funicular cord of *A. gelatinosa* (Fig. 7C).

#### Epistome and forked canal

**Fig. 11:**
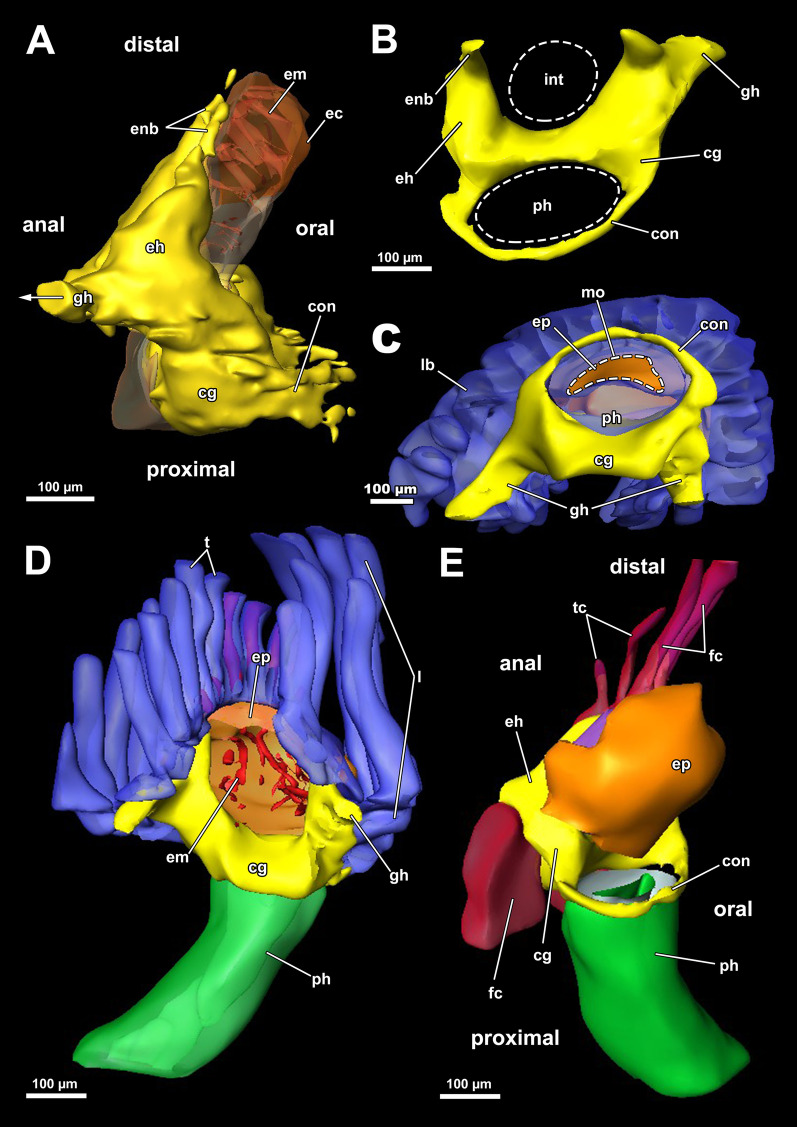
3D Reconstruction of the central nervous system and the lophophoral base of *Asajirella gelatinosa*. **A** Lateral view of the cerebral ganglion showing several projections: a circumoral nerve ring projects orally, epistomial horns ascend into the epistome and on the anal side ganglionic horns run into the lophophoral arms. **B**, **C** Distal view of the cerebral ganglion showing that it is located between pharynx and intestine and the circumoral nerve ring fully encloses the pharynx. **D** Anal view of the lophophoral base showing the epistome in front of the cerebral ganglion with traverse muscles projecting between the epistomial horns. The ganglionic horns project along the lophophoral arms. **E** Oblique frontal view on the lophophoral base showing the dome-shaped epistome in front of the cerebral ganglion, above the pharynx. The forked canal above the epistome supplies the tentacles of the lophophoral concavity. *cg* cerebral ganglion, *con* circumoral nerve ring, *ec* epistome coelom, *eh* epistomial horns, *em* epistome musculature, *enb* epistome neurite bundles, *ep* epistome, *fc* forked canal (tentacles), *gh* ganglion horns, *int* intestine, *l* lophophore, *lb* lophophoral base, *mo* mouth opening, *ph* pharynx, *t* tentacle, *tc* tentacle coelom

The epistome is located above the mouth opening as a thickened, ciliated epithelial protrusion over the pharynx (Figs. 4E, Fig. 5A, 6B). It is rather small and dome-shaped (Figs. 4E, 11E). The epistome is supported with a coelomic cavity that is connected to the remaining cavity by a narrow canal just above the cerebral ganglion (Figs. 4B–D, 6E). The cavity is spacious in the epistome, (Fig. 5C, F) and occasionally includes coelomocytes (Fig. 5E). Epistome muscles are either arranged as muscle fibres between the epithelial layers of the epistome to form an intraepithelial basket-like plexus (Figs. 5E, 10A–C) or as bundles proximo-distally traversing the epistomial coelomic cavity (Figs. 4A–D, 5, 10D). The muscular, intraepithelial basket of the epistome comprises only fine fibres, whereas the traversing muscles are much more prominent.

The forked canal is a ciliated, coelomic canal lining and supporting the three tentacles of the lophophoral concavity in *A. gelatinosa* (Figs. 4E, 5D–F, 11E). It emanates directly laterally of the cerebral ganglion and projects distally as a short canal (Fig. 5D). In contrast, the coelom of all other tentacles is unciliated.

### Nervous system

#### Central nervous system

**Fig. 12:**
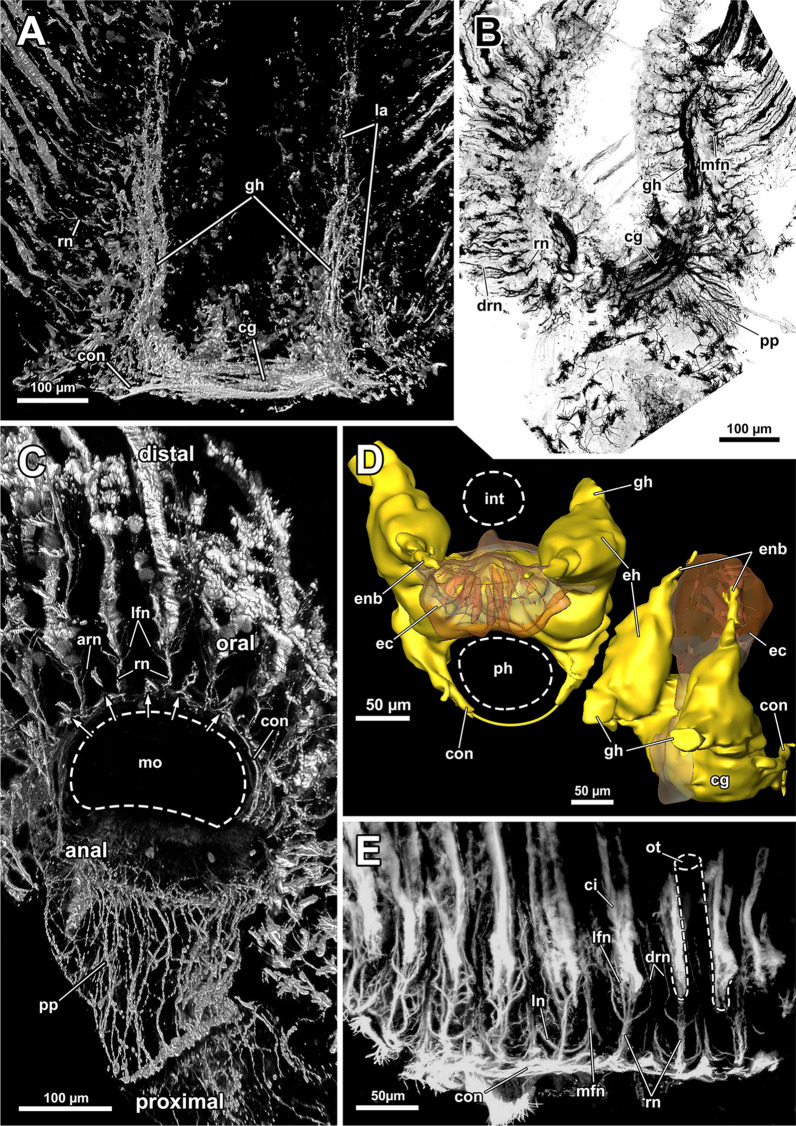
Overview of the central nervous system of *Asajirella gelatinosa* stained against acetylated-α-tubulin and 3D-recontruction of the cerebral ganglion. **A**, **B** Scans of the lophophoral arms showing the ganglionic horns projecting from the cerebral ganglion into the lophophoral arms. **C** Scan of the mouth opening and oral tentacles. A circumoral nerve ring surrounds the mouth opening. Proximally, a pharyngeal plexus is associated with the nerve ring. **D** 3D-reconstruction of the cerebral ganglion situated between pharynx and intestine showing several projections such as the circumoral nerve ring, the epistomial horns and the ganglionic horns. **E** Detail of the innervation of the oral tentacles showing the mediofrontal tentacle neurite bundles and the radial nerves branching off from the nerve ring. *arn* additional radial nerve, *cg* cerebral ganglion, *ci* cilia, *con* circumoral nerve ring, *drn* distal radial nerve, *ec* epistome coelom, eh epistome horns, *enb* epistome neurite bundles, *gh* ganglion horns, *int* intestine, *lfn* laterofrontal tentacle neurite bundle, *ln* lateral nerves, *mfn* mediofrontal tentacle neurite bundle, *mo* mouth opening, *ot* oral tentacles, *ph* pharynx, *pp* pharyngeal plexus, *rn* radial nerve

**Fig. 13:**
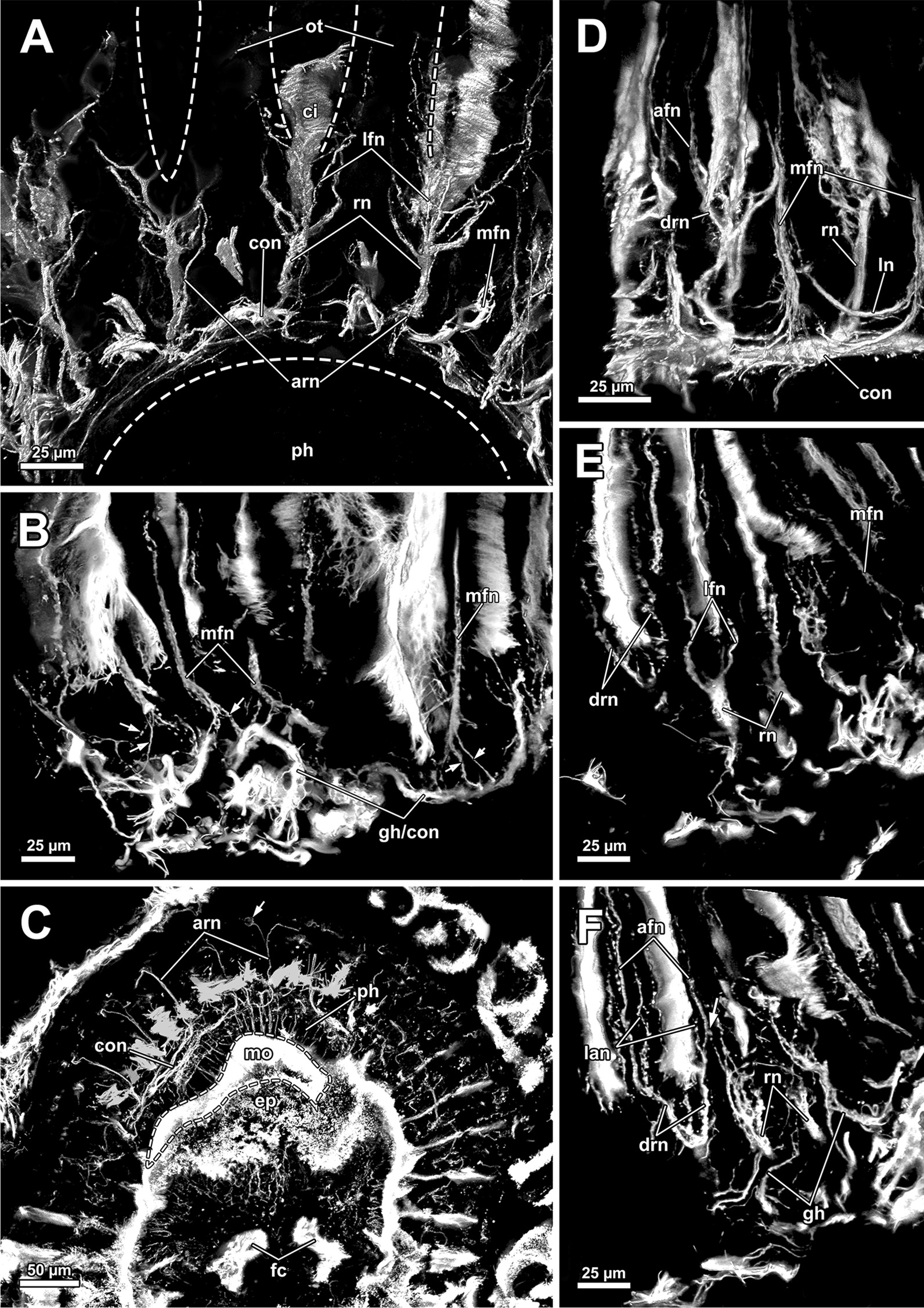
Details of the tentacle innervation in *Asajirella gelatinosa*, stained against acetylated-α-tubulin. **A** Radial nerves of the oral tentacles divert from the circumoral nerve ring intertentacularly. They branch in their distal traverse innervating adjacent tentacles. **B** A single mediofrontal nerve ascends into each tentacle as a series of thin rootlets (arrows) from the ganglionic horns or the circumoral nerve ring. **C** Vibratome section of the lophophore base showing thin, abfrontally bifurcating ‘additional radial nerves’. **D**–**F** Individual tentacle projections from frontal to abfrontal showing various tentacle neurite bundles branching off: Most frontally mediofrontal neurite bundles project from the circumoral nerve ring (**D**). Alternately, radial nerves project abfrontally with laterofrontal neurite bundles branching thereof (**E**). Abfrontally, the radial nerves bifurcate twice, resulting in lateroabfrontal and abfrontal neurite bundles (**F**). *afn* abfrontal neurite bundle, *arn* additional radial nerve, *ci* cilia, *con* circumoral nerve ring, *drn* distal radial nerve, *ep* epistome, *fc* forked canal, *gh* ganglion horns, *lan* lateroabfrontal neurite bundle, *lfn* laterofrontal tentacle neurite bundle, *ln* lateral nerves, *mfn* mediofrontal neurite bundle, *mo* mouth opening, *ot* oral tentacles, *ph* pharynx, *rn* radial nerve

The cerebral ganglion represents the center of the nervous system and is situated at the lophophore base between the pharynx region and the intestine (Fig. 12D). In *A. gelatinosa* the ganglion is of very large size and includes a considerable lumen (Fig. 6A, B, D, E). Neuronal tissue is located predominantly on the anal side, whereas the lumen occupies the oral side. Several projections emanate from the ganglion: on the anal side two ganglionic horns extend into the lophophoral arms (Figs. 12A, B, D, 11A–D). A circumoral nerve ring encompasses the pharynx on the lateral and oral side of the lophophoral base. From the circumoral nerve ring, radial nerves emerge into the tentacles and numerous neurite bundles branch off into the foregut to form a plexus of the pharynx (Figs. 11, 12C–E, 13A, C, E). The innervation of the lophophoral arm tentacles originates from the ganglionic horns (Figs. 12B, 13B, E, F). Two pyramidal extensions are located at the distal end of the ganglion and are herein referred to as epistomial horns (Figs. 4C–E, 11A, B, 12D). The epistomial horns include large lumina as well (Figs. 5A, 6A, C). In distal direction each horn becomes narrower and eventually projects as epistomial neurite bundle into the epistome.

#### Tentacle innervation

**Fig. 14:**
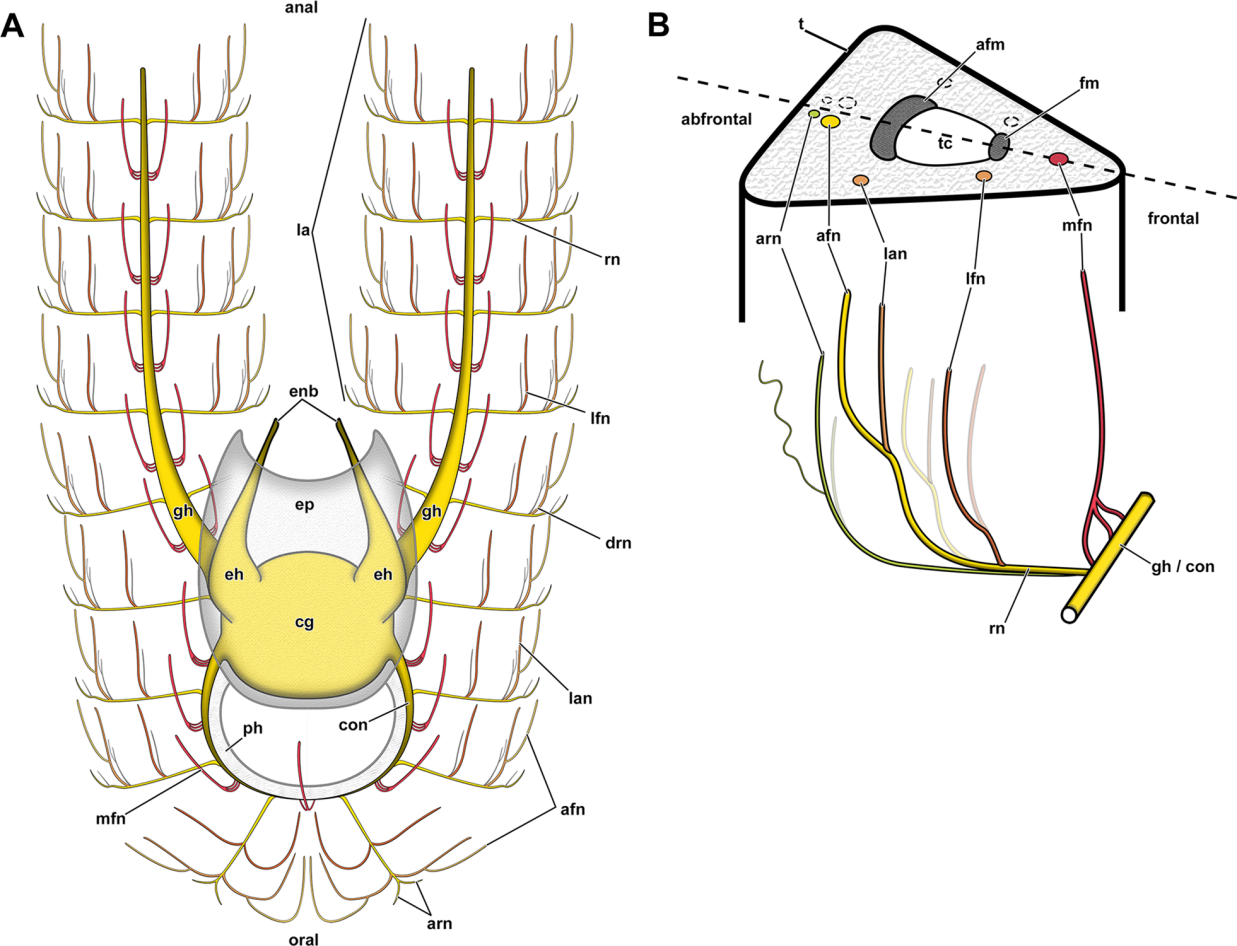
Schematic overview of the neuroanatomy of the lophophore base of *Asajirella gelatinosa*. **A** From the cerebral ganglion several projections extend: two ganglion horns into each lophophoral arm and the circumoral nerve ring around the foregut. Ultimately, two epistomial horns ascend distally and project as epistome neurite bundles into the epistome. **B** Schematic detail of the innervation of a single tentacle shows neurite bundles branching along the fronto-abfrontal axis: The radial nerve projects from the ganglionic horn/circumoral nerve ring abfrontally. Along the radial nerve, two laterofrontal neurite bundles branch off. Abfrontally, a pair of lateroabfrontal and abfrontal neurite bundles split off. Most abfrontally, the additional radial nerve branches off. Each tentacle is supplied from two adjacent radial nerves (lined circles). Frontally, a single mediofrontal tentacle neurite bundle project from the ganglionic horn/circumoral nerve ring into each tentacle. *afm* abfrontal tentacle muscle, *afn* abfrontal neurite bundle, *arn* additional radial nerve, *cg* cerebral ganglion, *con* circumoral nerve ring, *drn* distal radial nerve, *eh* epistomial horns, *enb* epistome neurite bundles, *ep* epistome, *fm* frontal tentacle muscle, *gh* ganglion horns, *la* lophophoral arm, lan lateroabfrontal tentacle neurite bundle, *lfn* laterofrontal tentacle neurite bundle, *mfn* mediofrontal tentacle neurite bundle, *ph* pharynx, *rn* radial nerve, *t* tentacle, *tc* tentacle coelom

The tentacle innervation is uniform throughout the entire lophophore and follows the same pattern. A radial nerve extends from the ganglionic horn or the circumoral nerve ring in between two tentacle bases from which several different neurite bundles branch off (Fig. 14). Each bifurcation of a radial nerve innervates two adjacent tentacles (Fig. 14B). The first pair of neurite bundles to branch off in its distal traverse are the laterofrontal neurite bundles (Figs. 12C, E, 13A, E). The radial nerve further bifurcates two times in distal direction. One of these branches continues as lateroabfrontal neurite bundle (Figs. 13E, F, 14), whereas the other projects as medio-abfrontal bundle (Figs. 13D–F, 14). At the bifurcation of the radial nerve, a slim neurite bundle, the additional radial nerve, ascends abfrontally into each tentacle base for a short distance. (Figs. 12C, 13C).

An additional tentacular neurite bundle, the mediofrontal neurite bundle, branches off directly as several thin rootles from the ganglionic horn, or the circumoral nerve ring and merge in the mediofrontal plane of each tentacle (Figs. 12B, C, E, 13A, B, D, E). In contrast to the intertentacular origin of all other tentacle neurite bundles, the mediofrontal neurite bundle rootlets emerge in the plane of a tentacle (Fig. 14B).

#### Gut innervation

**Fig. 15:**
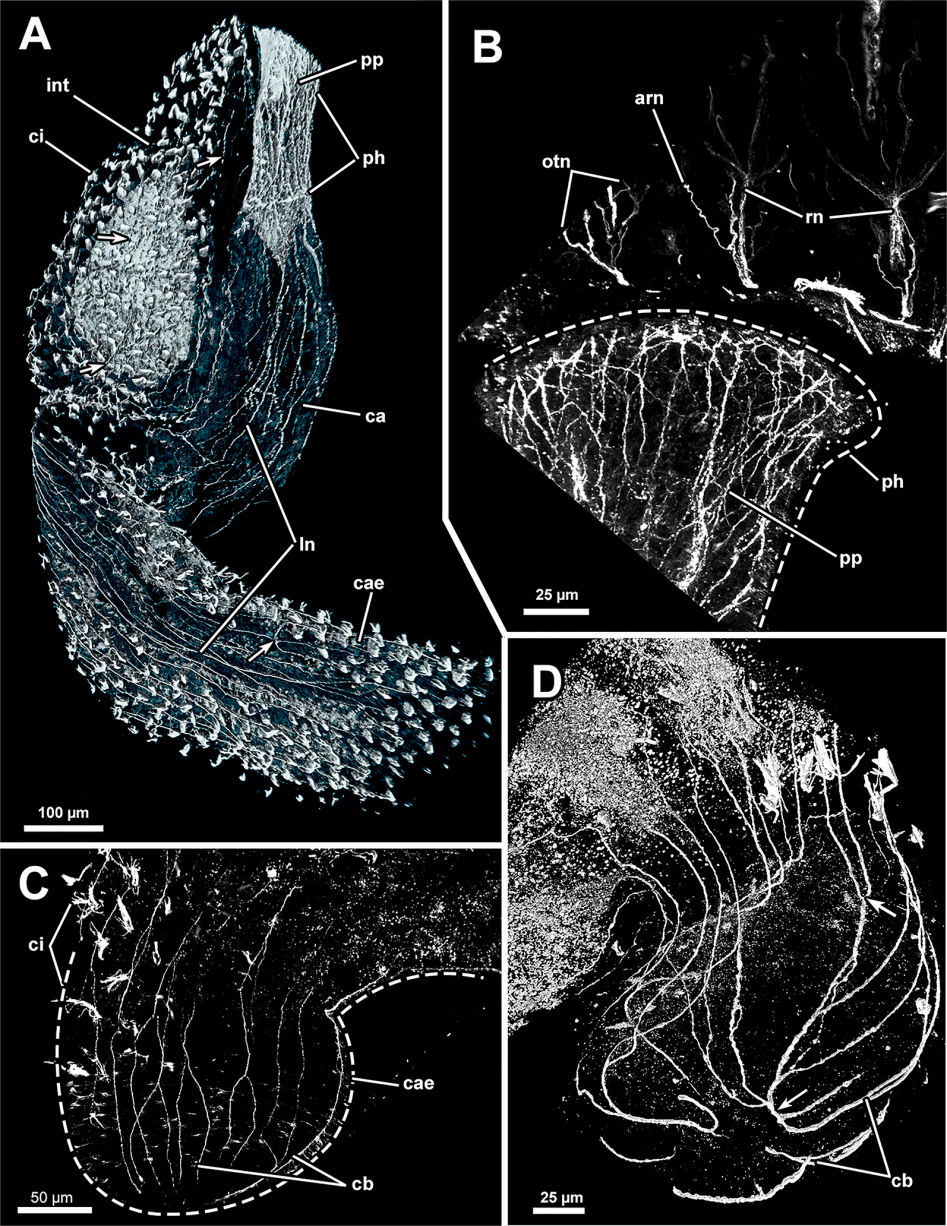
Peripheral nervous system of *Asajirella gelatinosa*, stainings against acetylated-α-tubulin. **A** Overview of the nervous system of the digestive tract showing a dense plexus in the pharynx and several longitudinal neurite bundles in the cardia and caecum. Few neurite bundles are present in the intestine (arrows). **B** Detail of the pharynx innervation showing dense plexus of longitudinally bifurcating neurite bundles. Tentacle innervation of the oral tentacles is also evident **C**, **D** Details of the proximal tip of the caecum showing a basket-like plexus of branching (**C**) and proximal fusing (**D**, arrow) longitudinal neurite bundles. *arn* additional radial nerve, *ca* cardia, *cae* caecum, *cb* caecum basket, *ci* cilia, *int* intestine, *ln* longitudinal neurite bundles, *otn* oral tentacle nerves, *ph* pharynx, *pp* pharyngeal plexus, *rn* radial nerve

Adjacent to the circumoral nerve ring, a nervous plexus extends proximally into the pharynx region. It comprises rather densely arranged, bifurcating longitudinal neurite bundles (Figs. 12B, C, 15A, B), whereas circular neurite bundles were not observed. Cardia and caecum show a less dense arrangement of, again, longitudinal neurite bundles (Fig. 15A, C, D). The neurite bundles at the proximal end of the caecum tend to fuse and bifurcate (Fig. 15C) and form a nervous basket around the caecum (Fig. 15D). A less dense neuronal network also exists in the intestine (Fig. 15A).

### Lophopodella carteri and *Lophopus crystallinus*

Only limited material was available for the other lophopodid genera. Since the representatives of both genera only differ in statoblast morphology, results are comparatively summarized for both species. We focus here on main differences found in those two genera when compared to *A. gelatinosa*.

#### General morphology

**Fig. 16:**
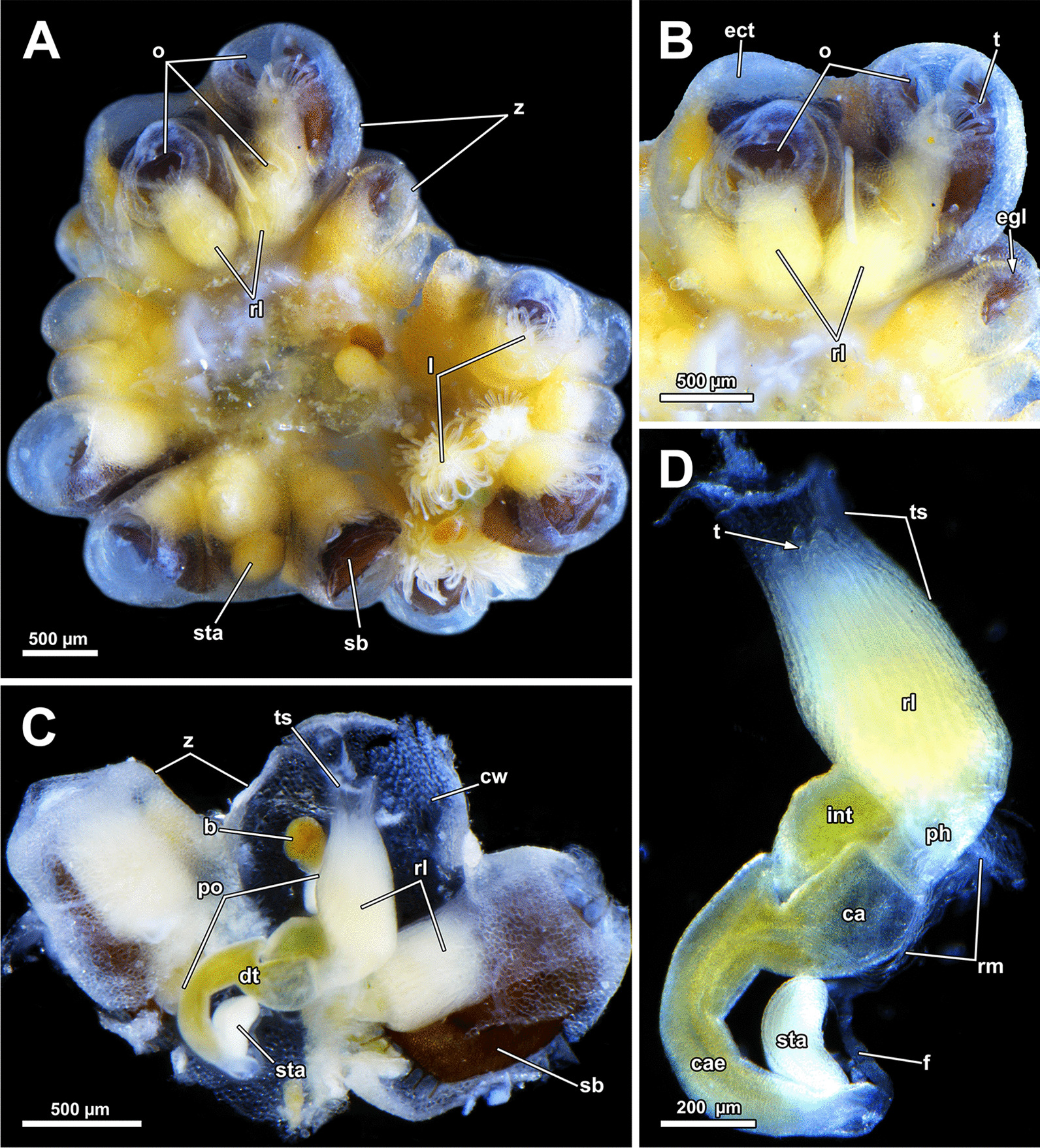
General overview of *Lophopodella carteri*. **A** Colony showing the circular and clustered arrangement of zooids including several mature statoblasts. **B** Detail of two retracted zooids. Epidermal gland cells are concentrated around the orifice and form a distal collar-like region. **C** Part of a colony showing a polypide with lophophore retracted into the tentacle sheath. **D** Overview of a sole polypide with retraced lophophore and the compartments of the digestive tract. *b* bud, *ca* cardia, *cae* caecum, *cw* cystid wall, *dt* digestive tract, *ect* ectocyst, *egl* epidermal gland cells, *f* funiculus, *int* intestine, *l* lophophore, *o* orifice, *po* polypide, *ph* pharynx, *rl* retracted lophophore, *rm* retractor muscles, *sb* statoblast, *sta* statoblast anlage, *t* tentacle, *ts* tentacle sheath, *z* zooid

In general, the investigated species of both genera do not form massive compound colonies as observed in *A. gelatinosa* but still grow into circular, clustered arrangements (Fig. 16A). On zooidal level the lophophore is less massive compared to *A. gelatinosa*, e.g. *L. carteri* has a mean lophophore hight of 0.57 mm (SD 0.051 mm) and a mean width of 0.37 mm (SD 0.055 mm; Fig. 16B, C) with a similar gross morphology (Fig. 16C, D). For *L. crystallinus* only sectioned material was available for this study, hence no measurement data is given.

### Body wall and aperture

**Fig. 17:**
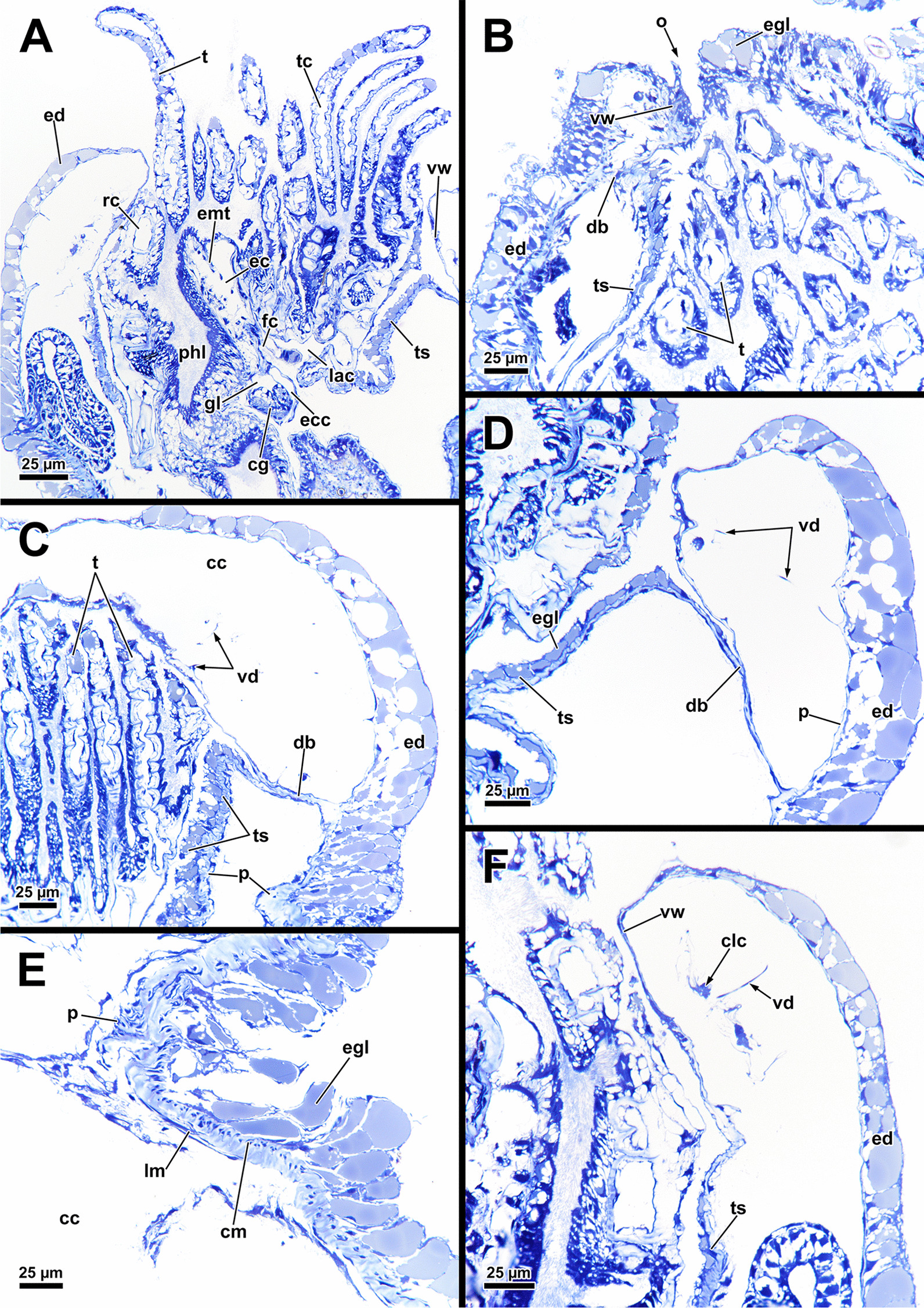
Histological details of the apertural area of *Lophopodella carteri*. **A** Lateral view of a half-retracted lophophore with the cerebral ganglion in the proximal region of the lophophore base. On the anal side, the epistome coelom canal, the forked canal, and the coelom of the lophophoral arms are visible. **B** Oblique section through the apertural area shows epidermal gland cells around the orifice. Compact duplicature bands connect the body wall and tentacle sheath. **C**, **D** Lateral view of the distal part of the zooid in retracted (**C**) and half-retracted condition (**D**). The tentacle sheath is rather thick (**C**) and includes several epidermal gland cells (**D**). Duplicatur bands connect the tentacle sheath to the body wall, vestibulum dilatators travers the coelomic cavity more distally to the duplicature bands, connecting the vestibular wall and body wall. **E**, **F** Histological details of the body wall and distal zooidal area. The epidermis includes vacuolated epidermal gland cells followed by a thin peritoneum. Several coelomocytes are seen in the distal zooidal area. *cc* coelom cavity, *cg* cerebral ganglion, *clc* coelomocytes, *cm* circular muscles, *db* duplicature bands, *ec* epistome coelom, *ecc* epistome coelom canal, *ed* epidermis, *egl* epidermal gland cells, *emt* transversal muscles of the epistome, *fc* forked canal, *lac* lophophore arm coelom, *lm* longitudinal muscles, *o* orifice, *p* peritoneum, *rc* ring canal, *t* tentacle, *tc* tentacle coelom, *ts* tentacle sheath, *vd* vestibulum dilatators, *vw* vestibular wall

**Fig. 18:**
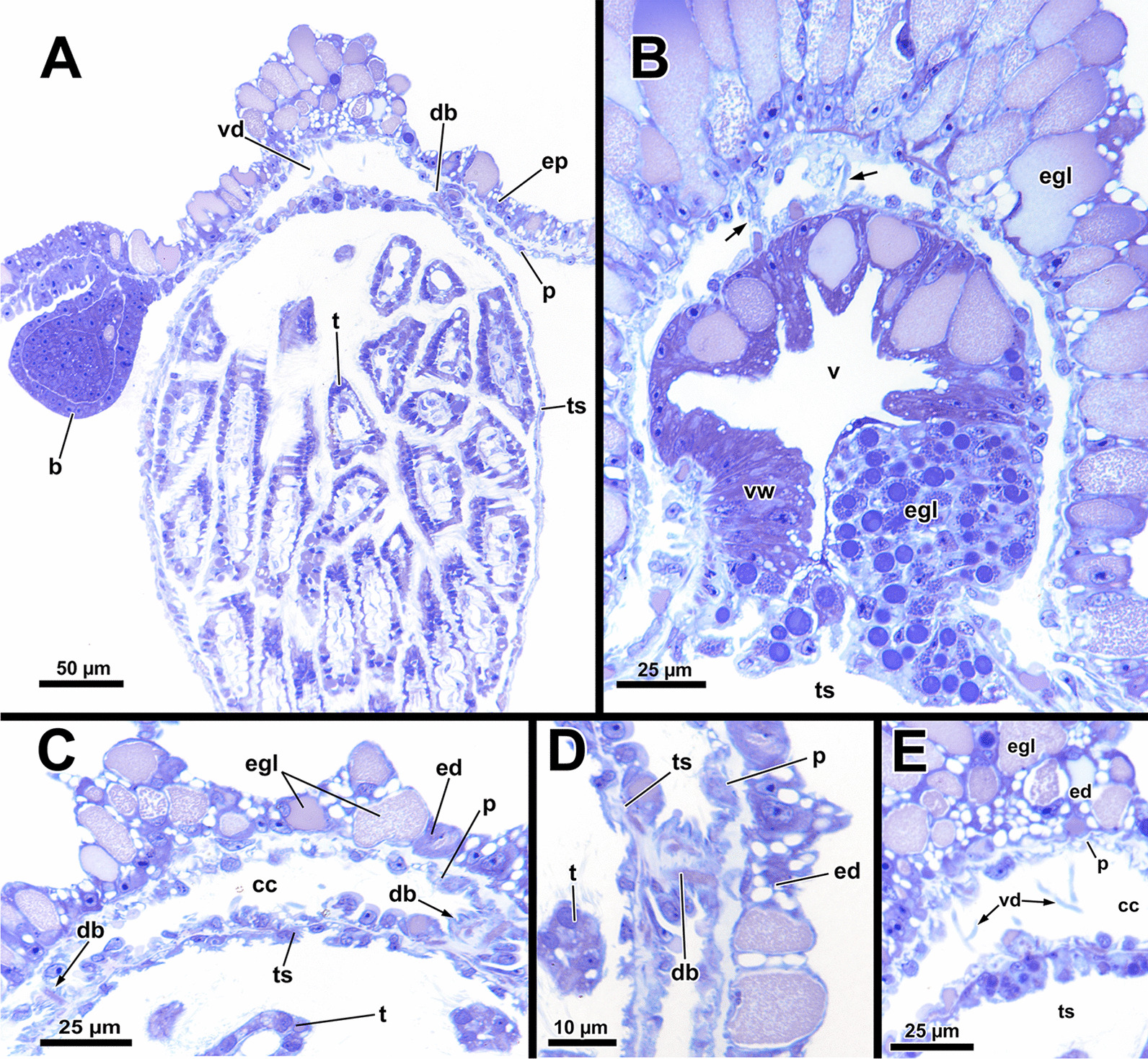
Histological details on the apertural area of *Lophopus crystallinus*. **A** Lateral section of a retracted zooid showing numerous epidermal gland cells in the distal epidermis, and vestibular dilatators and duplicature bands connecting the distal body wall to the tentacle sheath. **B** Detail of the vestibulum showing epidermal gland cells in the vestibular wall and numerous granular glands. **C**–**E** Thick duplicature bands connect the body wall with the tentacle sheath, and thin vestibular dilatators are located more distally. *b* bud, *cc* coelom cavity, *db* duplicature bands, *ed* epidermis, *egl* epidermal gland cells, *p* peritoneum, *t* tentacle, *ts* tentacle sheath, *vd* vestibulum dilatators, *ve* vestibulum, *vw* vestibular wall

The body wall shows an orthogonal grid of body wall muscles (Fig. 17E)*.* Epidermal gland cells with large vacuoles are present in the epidermis and are concentrated around the orifice (Figs. 17, 18). Vestibular wall glands were observed in *L. crystallinus*, but not in *L. carteri*. In the latter, the vestibular wall was not completely invaginated. All lophopodids show a similar situation concerning the apertural muscles. While the duplicature bands of *Lophopus* are similar to *Asajirella* (Fig. 18A, D), they are mostly elongated and slim in *Lophopodella* (Fig. 17C, D).

### Lophophore and tentacle morphology

**Fig. 19:**
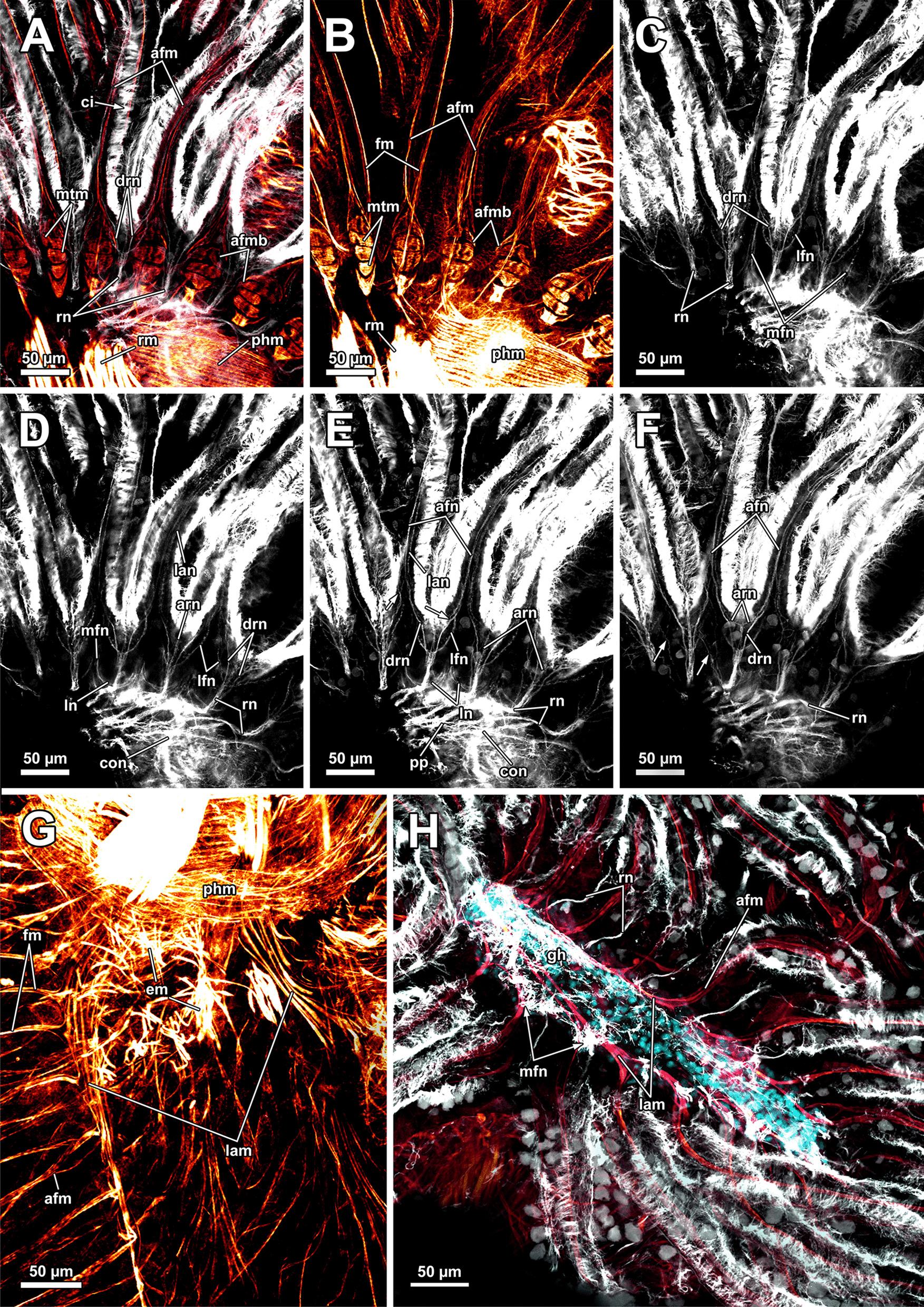
Neuromuscular details of the lophophore of *Lophopodella carteri*, stained for f-actin and against acetylated-α-tubulin. **A**, **B** Oral view of tentacle bases showing the intertentacular nerve origin (**A**) and muscle bases within the tentacles. The abfrontal muscle bases include at least 4 median transversal bands. **C**–**F** Details of the tentacle innervation, show a main radial nerve from which the laterofrontal (**C**), lateroabfrontal (**D**) neurite bundles and abfrontal neurite bundles (**E**) branch off and innervate one half of a tentacle. Abfrontal, very thin neurites ascent into the intertentacular membrane (**F**, arrows) Alternating with the radial nerves and therefore congruent with the tentacle muscles, a mediofrontal neurite bundle ascends individually and originates from the circumoral nerve ring (**C**, **D**). The pharyngeal plexus is associated with the proximal side of the circumoral nerve ring. Neighbouring mediofrontal neurite bundles are connected via lateral nerves (**D**). **G**, **H** Muscular (**G**) and neuronal (**H**) details of the lophophoral arms showing the tentacle muscles associated with the lophophore arm muscles and radial nerves emanating from the ganglionic horns. *afm* abfrontal muscle, *afmb* abfrontal muscle base, *afn* abfrontal tentacle neurite bundle, *arn *additional radial nerve, *ci* cilia, *con* circumoral nerve ring, *drn* distal radial nerve, *em* epistome musculature, *fm* frontal muscle, *gh* ganglionic horns, *lam* lophophore arm musculature, *lan* lateroabfrontal tentacle neurite bundle, *lfn* laterofrontal tentacle neurite bundle, *ln* lateral nerves, *mfn* mediofrontal nerve, *mtm* median transversal muscle, *phm* pharynx musculature, *pp* pharyngeal plexus, *rm* retractor muscle, *rn* radial nerve

Confocal microscopy data of *L. carteri* show slenderer lophophoral arms and also fewer tentacles (approx. 70), fortifying the large size of *Asajirella*. Nevertheless, the lophophoral arm musculature is comparatively pronounced and ramifies directly into the tentacle muscles (Fig. 19B, G, H). Although the abfrontal muscle bases of the lophophoral arms are as prominent as in *Asajirella* (Fig. 19A, G), the corresponding bases of the oral tentacles feature five median bands in *L. carteri* (Fig. 19A, B). *Lophopodella* samples show a high conformity with *Asajirella*: radial nerves branch off from the circum pharyngeal nerve ring or the ganglion horns (Fig. 19). At first laterofrontal neurite bundles bifurcate from the radial nerve (Fig. 19C–E), while the latter continues abfrontally before it ramifies into two distal branches of the radial nerve (Fig. 19A, C–F). Ultimately, each distal branch bifurcates again and a lateroabfrontal and abfrontal neurite bundle ascend each in a half of neighbouring tentacles (Fig. 19D–F). An additional radial nerve projects further abfrontal before it splits, and thin neurite bundles ascend for a short distance into adjoining tentacles (Fig. 19D–F).

A mediofrontal neurite bundle originates from the ganglionic horns and circumpharyngeal nerve ring (Fig. 19C–D). As occasionally encountered in *A. gelatinosa* (Fig. 12E), the mediofrontal neurite bundles in *L. carteri* are laterally connected to each other via lateral nerves (Fig. 19D, E). In contrast to *Asajirella*, thin neurites running parallel to the radial nerves were detected that extended into the intertentacular membrane (Fig. 19C, F).

### Lophophoral base and epistome

**Fig. 20:**
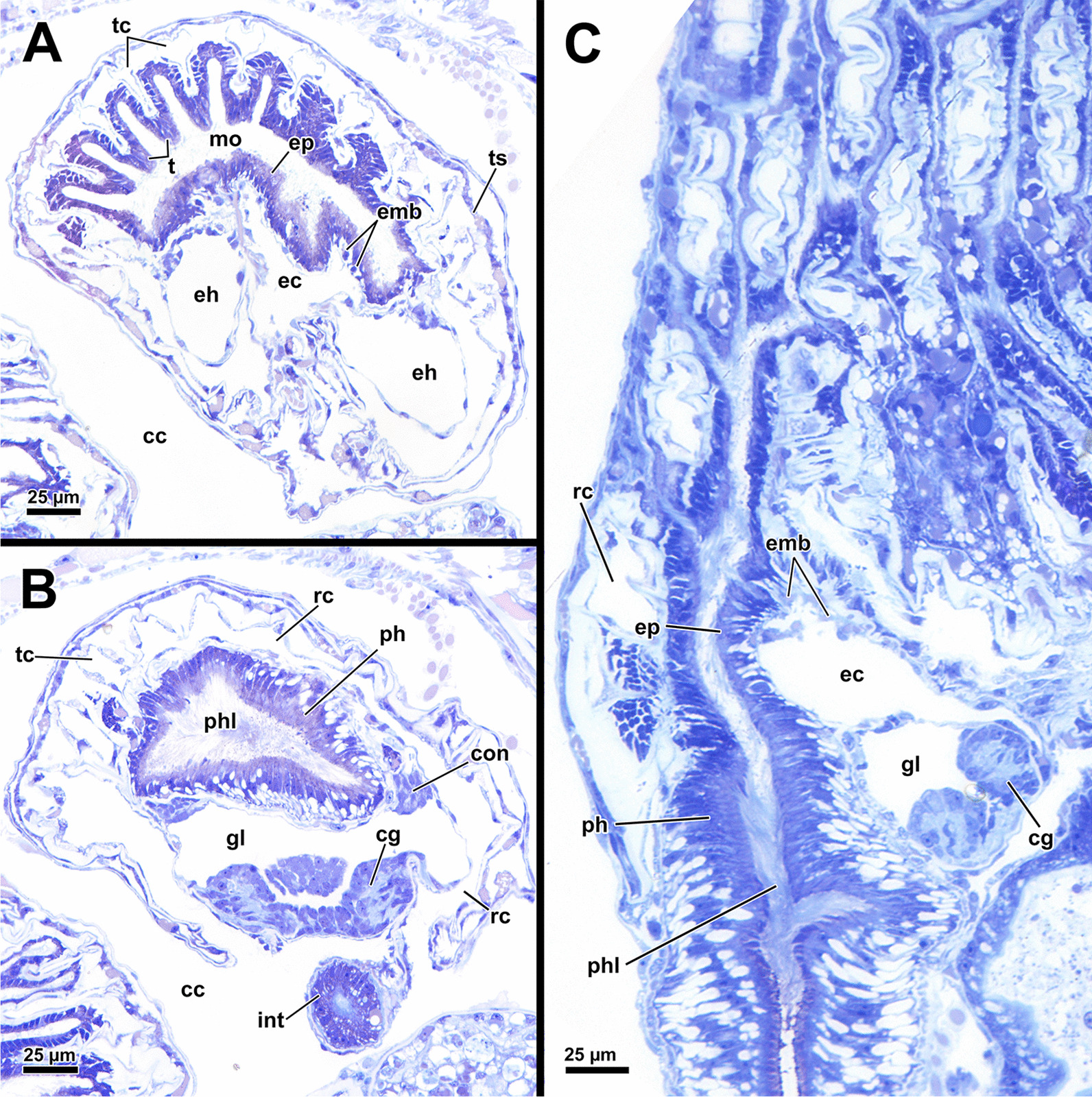
Histological details of the lophophoral base of *Lophopus crystallinus*. **A**, **B** Two different cross sections of the ganglion and epistome horns showing a large ganglion located between the pharynx the intestine. The ganglion and epistomial horns show a rather spacious lumen. **C** Sagittal section of the lophophoral base showing a small dome-shaped epistome. *cc* coelom cavity, *cg* cerebral ganglion, *con* circumoral nerve ring, *ec* epistome coelom, *eh* epistomial horns, *ep* epistome, *gl* ganglion lumen, *int* intestine, *mo* mouth opening, *ph* pharynx, *phl* pharynx lumen, *rc* ring canal, *tc* tentacle coelom

**Fig. 21:**
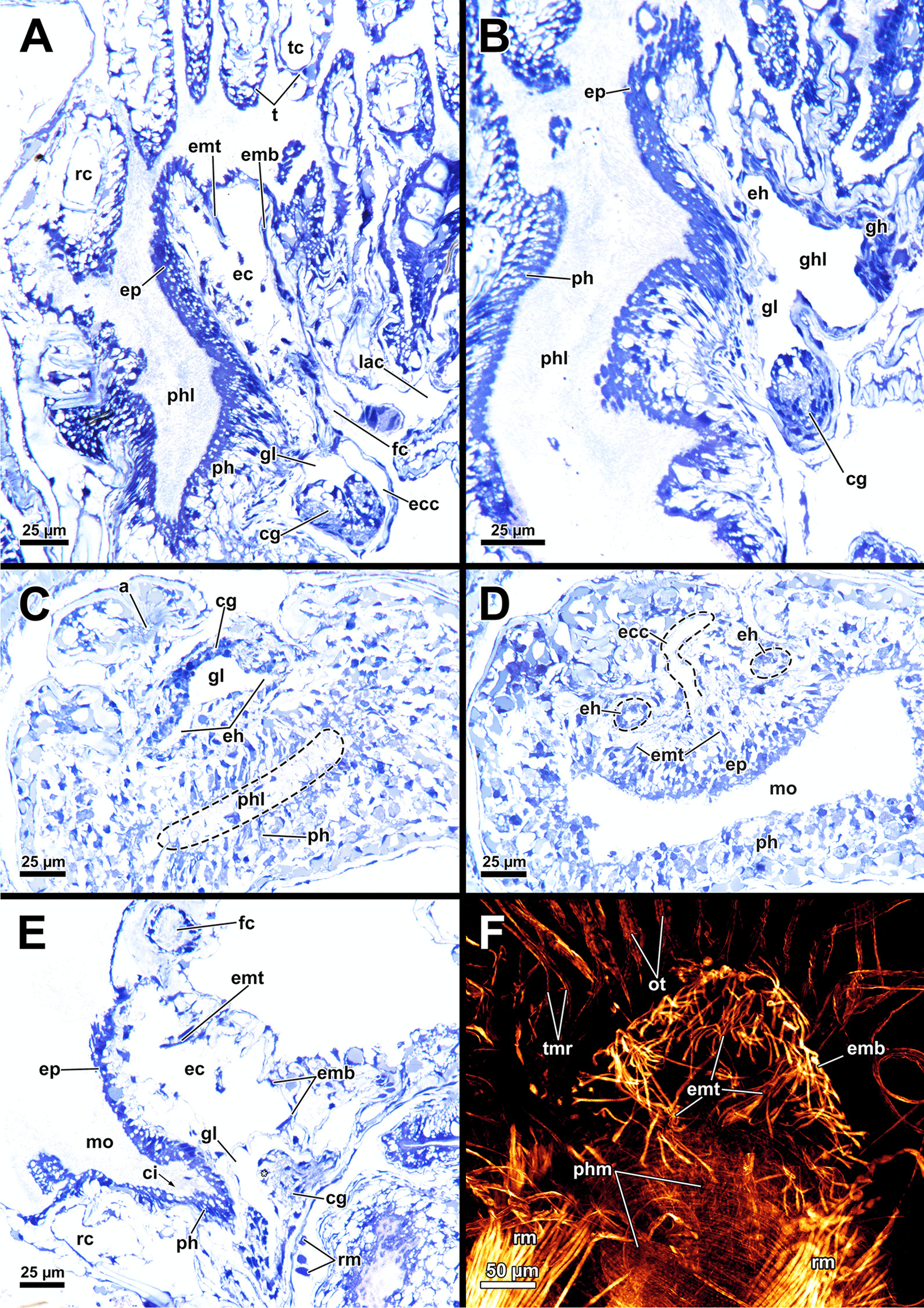
Details of the epistome of *Lophopodella carteri*. **A**–**D** Longitudinal (**A**, **B**) and cross (**C**, **D**) sections of the lophophoral base showing a comparatively small, dome-shaped epistome with transversal epistome muscles within its coelomic cavity. Muscles are also embedded in its epithelial linings (**A**). Two prominent epistomial horns extend from the cerebral ganglion (**B**) and ascend laterally of the epistome coelom canal (**C**, **D**). In the ganglion and the epistomial horns, an extensive lumen is evident (**B**). **E** Lateral view from the lophophoral base showing the cerebral ganglion located beneath the epistome and the ciliated forked canal supplying a tentacle of the lophophoral concavity. **F** Anal projection of the epistome musculature stained for f-actin showing transversal and basket like epistome muscles and a pair of retractor muscle bundles inserting at the lophophoral base, anally of the epistome. *a* anus, *cg* cerebral ganglion, *ci* cilia, *ec* epistome coelom, *ecc* epistome coelom canal, *emb* epistome muscle basket, *emt* transversal muscles of the epistome, *ep* epistome, *fc* forked canal, *gl* ganglion lumen, *gh* ganglionic horns, *ghl* lumen of the epistome / ganglion horns, *lac* lophophore arm coelom, *mo* mouth opening, *ot* oral tentacles, *ph* pharynx, *phl* pharynx lumen, *rc* ring canal, *rm* retractor muscles, *t* tentacle, *tc* tentacle coelom, *tmr* tentacle muscle roots

Since the lophophoral base represents the most complex area of the zooid it is of particular interest to investigate. As in *Asajirella*, the epistome is a dome-shaped protrusion (Figs.17A, 20A, C, 21A, B, D). The epistome of both, *L. carteri* and *L. crystallinus* feature the same two sets of muscles: a muscle basket embedded into the epistome (Figs. 20A, C, 21A, E, F) and more pronounced muscle bundles transverse the epistome coelom (Figs. 17A, 20A, C, 21). Beside the circumoral nerve ring and the ganglionic horns, two epistomial horns project from the cerebral ganglion distally into the epistome (Figs. 20A, 21B, D). Although the epistomial horns of all lophopodids feature a lumen, the latter seems less pronounced in *L. carteri* (Fig. 21D). The anatomy of the cerebral ganglion itself is identical to *Asajirella* in respect to the large size and corresponding lumen (Figs. 20, 21A–C, E).

### Neuromuscular system of the digestive tract

**Fig. 22:**
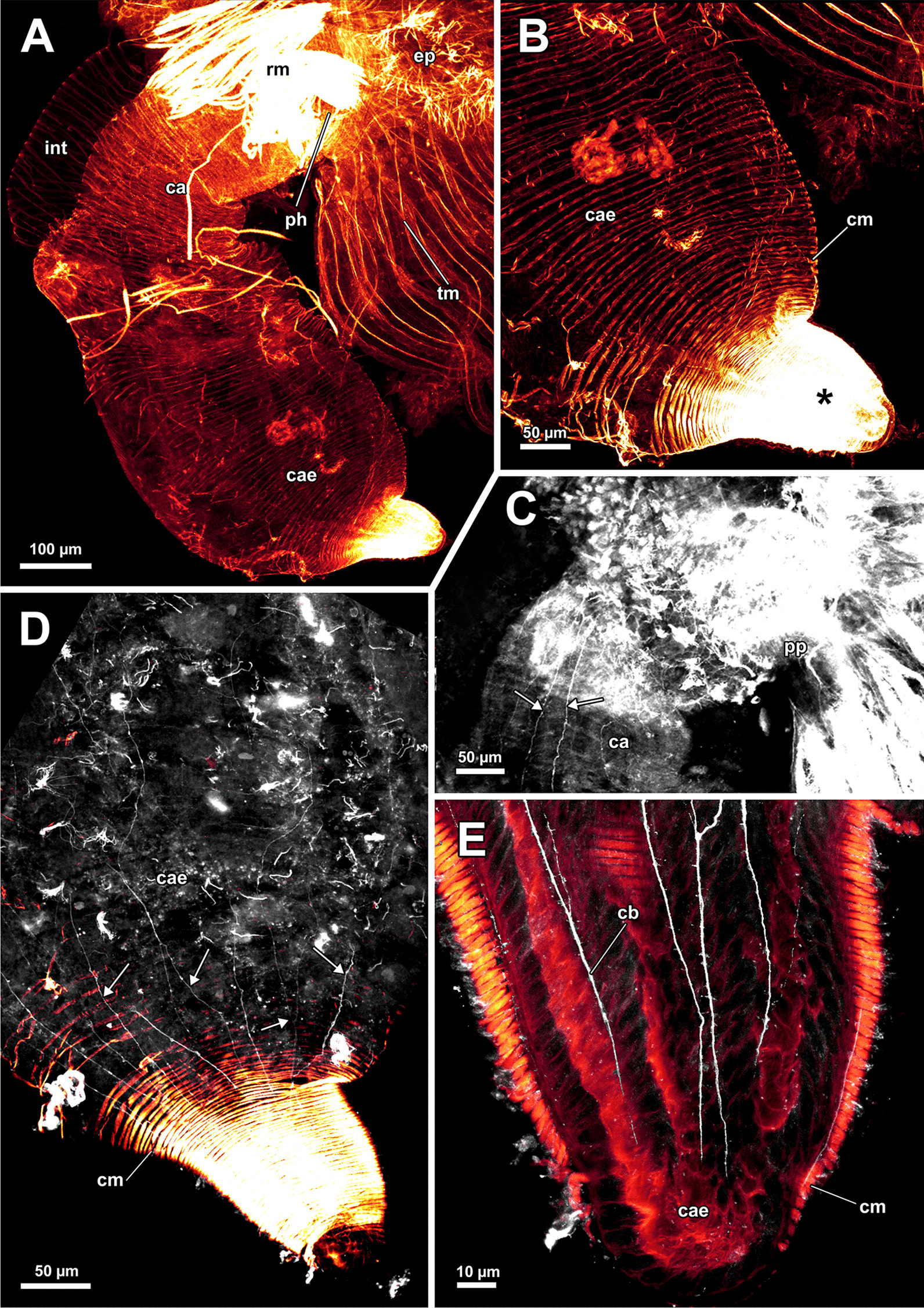
Neuromuscular system of the digestive tract of *Lophopodella carteri*, stained for f-actin and acetylated-α-tubulin. **A** General myoanatomy of the digestive tract showing exclusively circular musculature in the digestive tract with dense arrangement in the pharynx and only few circular muscles in the intestine. **B** The proximal end of the caecum is very contracted and therefore also shows a very intense signal (asterisk). **C** The nervous system of the digestive tract includes a dense plexus in the pharynx region, few longitudinal neurite bundles in the cardia (arrows) and almost no staining in the intestine. **D**–**E** The caecum features a diffuse network of thin longitudinal neurite bundles (**D**, arrows). The proximal tip of the caecum includes a caecum basket in the form of comparable prominent longitudinal neurites as well (**E**). *ca* cardia, *cae* caecum, *cb* caecum basket, *cm* circular muscles, *ep* epistome, *int* intestine, *ph* pharynx, *pp* pharyngeal plexus, *rm* retractor muscles

The data on *L. carteri* show that the digestive system includes exclusively circular muscles, that are most dense in the pharynx and in the proximal end of the caecum (Fig. 22A–C, E). Concerning the peripheral nervous system, the highest number of neurite bundles was detected in the pharynx (Fig. 22D). Additional longitudinal neurite bundles were also detected in the cardia and especially the caecum, although not as clear as in *Asajirella* (Fig. 22C–E). This also accounts for the nervous plexus at the proximal end of the caecum (Fig. 22E).

## Discussion

### General morphology

There are several comprehensive studies on the colony morphology of lophopodids (*Lophopus* / *Lophopodella*: [9, 27, 30, 31, 40, 41]; *Asajirella*: [31–33]) which show similar results as the present study. Colonies consist of small circular arranged zooids. They reach a diameter up to 2.5 cm and often form massive compound colonies [42, 43]. As reported for *Lophopus* [44], also *Asajirella* was found to grow on plant substrates such as leaves and submerged wood. The cystid walls of *A. gelatinosa* show no septation of any sort between zooids [32, 33]. In contrast to other gelatinous families, the digestive tract of *A. gelatinosa* remains straight and unfolded in retracted condition as described by previous authors [32]. Between functional zooids, small protrusions represent empty cystids with degenerated polypides [32].

### Body wall/cystid

Previous histological investigations demonstrated conspicuous large cells in the epidermis of the body wall, which are most likely responsible for the mamillated appearance of the cystid described above [35, 37]. In addition, these cells are concentrated at the distal end around the orifice and show large vacuoles with amorphous content. The content of this cells is proteinaceous, with phospholipid components and stains PAS-negative [35]. The chemical composition of the vacuoles varies in dependency of e.g. the developmental stage of the latter [35]. Similar vesicular cells are reported for *Lophopodella carteri* [30, 37] and *Lophopus* [27, 44] and are generally described as vacuolar cells (“Blasenzellen”). Sections of *Lophopodella* and *Lophopus* in this study confirm their presence in all lophopodids. Other epidermal cells lacking large vacuoles are located in between these cells. These cells contribute to the secretion of the gelatinous ectocyst [27, 30, 44] and are also seen as precursor of the ectocyst [35]. Besides lophopodids, *Cristatella mucedo* and *Pectinatella magnifica* also show similar cells, although there they are restricted to the basal side of the colony and vestibular wall [45, 46]. In addition to the mentioned epidermal cells, distinct glands, generally referred to as vestibular glands (“white spots”) exist on the anal side of the duplicature (vestibular wall) in *P. magnifica*, *L. carteri* and *L. crystallinus* and do not participate in the secretion of the ectocyst [25, 37, 44]. Vacuolar cells are also known in Plumatellidae and Fredericellidae [46–49]. In summary, epidermal gland cells including large vacuolar ones are present in all phylactolaemates (for Stephanellidae see [23]) and most pronounced in families with gelatinous ectocysts (Lophopodidae, Pectinatellidae, Cristatellidae). Concentrations around the orifice are common in lophopodids and found in Cristatellidae as well.

### Myoanatomy

#### Musculature of the body wall

In general, the body wall musculature of phylactolaemates comprises a regular grid of outer circular and inner longitudinal muscles embedded in the extracellular matrix of the epidermis and peritoneum [14, 19, 20, 23, 50]. The circular muscles are associated with the former and the longitudinal muscles with the latter [50][50]. At least in *Stephanella hina*, the peritoneal longitudinal muscles are more prominent [23]. *Lophopus crystallinus* and *Pectinatella magnifica* possess an additional layer of diagonal muscles in some areas of the body wall [14, 27]. In *A. gelatinosa* only two layers of body wall musculature are present. Owing to dissection of most zooids in preparation for confocal laser scanning microscopy, mostly vibratome sections allowed proper investigations of the body wall at the distal end, where a third layer of muscles is present in *Pectinatella magnifica*. However, no signs of a third layer of body wall muscles were detected in *A. gelatinosa*, neither in the distal nor in the lateral areas, similar to *Lophopodella carteri* (see also [30]). Thus, the only reported lophopodid featuring three layers of body wall muscles is *Lophopus crystallinus*, where they occur mainly in basal and lateral areas [27]. In general, all available data suggest a bi-layered body wall musculature to the be the plesiomorphic condition and a third layer evolved twice independently.

#### Apertural muscles and tentacle sheath

In retracted condition, the vestibular wall continues proximally into the tentacle sheath. The former being the continuation of the body wall also includes a grid of circular and longitudinal muscles [14, 50]. Longitudinal muscles of the vestibular wall are reported to be more prominent [50], whereas the present study finds pronounced circular muscles in *A. gelatinosa*, especially dense around the orifice and longitudinal ones are scarce. Data of the most basal branching Stephanellidae indicate both, circular and longitudinal musculature as prominent [23]. At the transition of the vestibular wall to the tentacle sheath a diaphragmatic sphincter muscle is present in most bryozoans [5, 14, 16, 23]. Similar to previous reports on the apertural musculature of phylactolaemates (see citations above), the sphincter muscle in *A. gelatinosa* and *L. crystallinus* is hardly differentiable from the circular muscle of the vestibular wall.

Large columnar epidermal cells in the proximal vestibular wall stain intensely in *A. gelatinosa* and feature large vacuoles in the distal tentacle sheath in *L. carteri*. Previous investigations found large vacuoles especially concentrated in the distal region of the body wall around the orifice in *L. carteri, L. crystallinus, A. gelatinosa, P. magnifica* and *C. mucedo* [37]. All those investigated species possess vacuolar cells of columnar to cubic shape in the vestibular wall and tentacle sheath as well, whereas in *C. mucedo* they are most abundant [35, 37]. *L. crystallinus* possesses a distinctive vestibular gland located on the anal side of the body wall [25].

The remaining apertural muscles of all phylactolaemates include two further sets: vestibulum dilatators and duplicature bands [5]. The former are exclusively single smooth muscles [6] that traverse the distal tip of the coelomic cavity radially and interconnect the lateral body-wall with the vestibular wall [14, 50]. This is also true for *A. gelatinosa*. The second set of muscles, the duplicature bands are peritoneal bands that extend from the body wall to the tentacle sheath [5, 50]. Only lophopodids were regarded as exceptional with the duplicature bands inserting directly at the sphincter muscle [27, 30, 51, 52]. The present study shows that the insertion of the duplicature bands in *A. gelatinosa*, but also in *Lophopus crystallinus* and *Lophopodella carteri* is located at the tenacle sheath and not at the diaphragmatic sphincter as previously indicated (see above citations). These data not only prove this character to be common for lophopodids, but a phylactolaemate and even bryozoan one.

The tentacle sheath is a continuation of the vestibular wall and in retracted condition enwraps the lophophore in a cavity termed atrium [5, 6, 50]. Recent studies showed significant differences in phylactolaemate tentacle sheath musculature by CLSM data [14]. In general, the tentacle sheath includes a rather fine structured mesh of orthogonal muscles. In *Lophopus crystallinus* [27] and *A. gelatinosa* [6] mostly longitudinal muscles are reported based on light- (*L. crystallinus*) and transmission electron microscopy (*A. gelatinosa*). New f-actin stainings and confocal analysis of the present study confirm the strongly pronounced longitudinal muscles and no indications of circular muscles. In addition, the former appears to branch in the lophophoral base area in *A. gelatinosa*. In *L. carteri*, only longitudinal muscles are depicted in the tentacle sheath as well [25] Hence, the branching, traverse fibres could so far only be verified for *A. gelatinosa*, but not any other lophopodid. The lack of circular musculature supports previous findings in *L. crystallinus* [27] and accordingly corroborates it as a general feature of this family. In contrast to lophopodids, circular muscles are present in the tentacle sheath of Cristatellidae, Pectinatellidae and Fredericellidae [14, 16]. Plumatellids possess circular muscles only close to the lophophoral base [14, 21] where the branching longitudinal muscles were found in *A. gelatinosa* (this study). Ultimately, the tentacle sheath of Stephanellidae includes an orthogonal grid of longitudinal and circular muscles [23]. This indicates it to be the ancestral situation.

#### Lophophore and digestive tract

The lophophore of all phylactolaemates features several sets of muscles, including the lophophore arm musculature and the muscles of the epistome [14]. In all phylactolaemates several muscle fibres extend from the lophophoral base into the arms of the horseshoe shaped lophophore [50] and enable high flexibility of the latter [21]. The lophophoral arm musculature is generally more pronounced in larger species such as *Pectinatella magnifica* and *Cristatella mucedo*, when compared to more slender representatives such as plumatellids [14]. Two muscles ascend in each lophophoral arm tentacle. Abfrontally, the muscles possess a large base with several traverse median bands in Cristatellidae, Pectinatellidae, and several plumatellids [14, 16, 21]. This is different in Stephanellidae. Instead of median bands, *Stephanella hina* possesses several stacked, spindle shaped longitudinal muscles [23]. The amount of muscle bands in the investigated families and species seem to vary from three to five, however no indication on the significance of this observation is present so far. Probably it correlates with polypide and thus lophophore size. The second longitudinal muscle ascending into each tentacle is situated frontally. However, the bases of the frontal tentacle muscles merely consist of two to three muscle fibre rootlets that adjoin and ascend into each tentacle [14]. In *A. gelatinosa* the frontal muscle of the lophophoral arm tentacles originate from two rootlets that branch off from the lophophoral arm muscles. The abfrontal muscle base connection to the lophophoral arm muscles is also present in other representatives with large lophophores e.g. Pectinatellidae, Cristatellidae [14]. On the contrary, *Stephanella* possess only a proximal extension to the muscles of the lophophoral arms [23] and in plumatellids such as *H. punctata* even a gap is present [14]. The observations of this study on *Lophopodella carteri* indicate that lophopodids in general have their tentacle muscles connected to the lophophoral arms. The cavities of the oral tentacles of cristatellids, pectinatellids and plumatellids are proximally bordered by the wall of the ring canal, whose radial muscles extend from the pharynx musculature [50]. In addition, frontal tentacle muscles of *Cristatella mucedo* have three oral rootles and show lateral interconnections. Oral tentacles do not differ from tentacles of the lophophoral arms in *A. gelatinosa*. No ring canal muscles were observed. Lastly, the tentacles of the lophophoral concavity include slender frontal muscles in connection with the epistome muscles. The three tentacles in the lophophoral concavity of *A. gelatinosa* are supplied by the heavily ciliated forked canal of the coelom. These observations are in accordance with findings in *Cristatella mucedo* and *Pectinatella magnifica*, in which at least the frontal tentacle muscles of the lophophoral concavity are associated with epistome muscles [14].

The digestive tract of all phylactolaemates consists of a pharynx and oesophagus that are separated from the following cardia by a valve that hinders reflux of food from the midgut. The cardia is followed by a large caecum that opens via the pylorus into the intestine [5, 50]. A layer of circular muscles surrounds the gut [5, 14, 23, 27]. Solely in *A. gelatinosa* longitudinal muscles were mentioned [6], which would be unique among phylactolaemates. Contrary to this previous claim, the present study clearly demonstrates the lack of such muscles. Apart from that the digestive tract musculature of lophopodids shows no conspicuous differences from other described phylactolaemates. From the proximal end of the caecum, the funiculus includes peritoneal longitudinal muscles and helps anchoring the digestive tract. This is the case in *A. gelatinosa* (this study), *L. carteri* [30], *L. crystallinus* [27] and all other phylactolaemate families [4, 14] except for Stephanellidae [23].

#### Epistome

Two different types of muscles can be found in the epistome: the first represents a muscular, intraepithelial basket present in the epistomial wall of Cristatellidae, Fredericellidae, Plumatellidae [14, 16, 21] and partially in Stephanellidae [23]; the second includes individual, thick muscle fibres traversing the epistomial cavity. They stretch from the oral epistome wall anally above the cerebral ganglion, where the epistome coelom ascends from the remaining coelomic cavity. The latter configuration is found in Stephanellidae, Pectinatellidae and the plumatellid *H. punctata* [14, 23] as well as *Lophopus* [27] and *Lophopodella* [30]. *A. gelatinosa*, has both types of epistome musculature. Rather thin muscles are embedded into the oral epistome wall and additionally thicker muscle fibres project from the epistome wall anally. This has so far only been observed in the plumatellid *H.* punctata ([14], for *S. hina* also see [23]). This study further shows that *Lophopodella carteri* also possesses a muscular, intraepithelial basket that generally seems less pronounced than in *A. gelatinosa.* Thus, the data of the present study show that traversal epistome muscles exist in all lophopodids and also the presence of a muscular, intraepithelial basket in the whole clade is assumed.

### Nervous system

#### Central nervous system and tentacle innervation

In all phylactolaemates the central nervous system consists of the cerebral ganglion and its extensions. The former is a neuroepithelium and originates during budding from the inner budding layer by invagination [19, 39, 52]. Throughout the whole taxon, the ganglion represents a neuroepithelial vesicle with a fluid-filled lumen and neuropil/somata in the periphery [13, 15, 19, 22, 23, 26, 46]. Early analyses showed the lumen of the ganglion to be rather wide and spacious [13, 27, 51, 53]. Several of the referred reports described large fixation artefacts, making the spacious lumen of the ganglion artificial [19]. In fact, all recent analyses show a rather small ganglion lumen in Stephanellidae [23], Cristatellidae [15], Fredericellidae and several Plumatellidae [20, 22]. Consequently, the large lumen of the ganglion in all lophopodids found in the present study represents a lophopodid specific trait that was rightfully depicted in some previous analyses of *L. crystallinus* [24, 27, 53]. With the exception of *Lophopodella carteri*, the pyramidal epistomial horns confirmed for all lophopodid genera in the present study have not been described in any other phylactolaemate. In *L. carteri* the “epistomial nerve ring” (epistomial neurite bundles) becomes wider as it approaches the cerebral ganglion and thereby features two “neural cavities” which correspond to the lumen of the epistomial horns of the present study [30]. Thus, the epistome horns found in the present study constitute another apomorphy of lophopodids. Concerning the tentacle innervation, no significant variation from the ground pattern was detected. In *C. mucedo, F. sultana, P. repens* the lateral nerves branching off the radial nerve appear to be taxon-specific [18]. The medio frontal neurite bundles of the early branching *S. hina* emerges from several rootlets, as found in the lophopodids of this study and recently in *C. mucedo* contrary to previous results [16]. However, the rootlets show several lateral connections in *S. hina* [23]. This study confirms lateral nerves in several specimens, but they solely connect neighbouring mediofrontal neurite bundles without any association to the radial nerve. Since the lateral nerves were only occasionally encountered and their amount was not consistent hardly any indications about their taxon specificity are possible.

#### Innervation of the tentacle sheath and digestive tract

In all investigated phylactolaemates, the tentacle sheath generally features a diffuse nerve plexus [13, 15, 18, 22, 23, 27]. This plexus originates from several tentacle sheath nerves branching directly from the cerebral ganglion [13, 22] and the circumoral nerve ring (see [18]). Although the plexus of the tentacle sheath is generally referred to as diffuse, it includes perpendicular arranged neurite bundles in *C. mucedo* [18] and thus features circular and longitudinal neurites. Only longitudinal neurite bundles were detected in the tentacle sheath of *A. gelatinosa* similar to the plumatellid *H. punctata* [22]. Several longitudinal neurites merge at the base of the tentacle sheath and thereby give the impression of ramifications in both species.

Similar to the tentacle sheath, the visceral innervation observed in the current study shows the same pattern as in other investigated species (see citations above). A plexus originates from the proximal side of the ganglion and the circumoral nerve ring [13, 18, 22, 23]. This plexus is most dense in the pharynx until the cardiac valve and shows only few longitudinal neurite bundles in the cardia. Circular bundles are present in the caecum of *S. hina* [23] and *H. punctata* [22] and are associated to the circular musculature of the latter. In addition, a dense plexus of circular neurites exists also between the epidermal and peritoneal layer of the caecum in *C. mucedo* [15]. In lophopodids only longitudinal bundles, but no circular ones, were observed in the caecum. At the proximal end pronounced longitudinal neurite bundles form a plexus by ramification. Only a thin nervous plexus was detected in the intestine of *A. gelatinosa*, similar to other phylactolaemates with the exception of *S. hina,* for which no innervation was found in the intestine [23].

## Conclusions

This study provides the first modern, comprehensive morphological analysis of the neuromuscular system of lophopodid phylactolaemates, including new data on the morphology of *A. gelatinosa*. Lophopodids feature some unique, apomorphic characters, which include a small, dome like epistome, pyramidal protrusions of the cerebral ganglion (epistomial horns), and a large ganglion with an extensive lumen. This study also corrects previous reports of longitudinal muscles in the digestive tract of *A. gelatinosa*, and clarifies that the digestive tract of all phylactolaemates comprises solely circular musculature. Interestingly, the early branching lophopodids show transversal, intracoelomic and intraepithelial muscles in the epistome, which has so far only been found in *Hyalinella punctata* of the later-branching plumatellids. Further details of the musculature, as the duplicature bands inserting at the tentacle sheath and not the diaphragmatic sphincter, contrary to previous reports support this feature as part of the ground pattern of phylactolaemates. Ultimately, this study fills the gap of modern morphological analyses for the last phylactolaemate family for which data were missing.

## Data Availability

The datasets produced and/or analysed during the current study are available from the corresponding author on reasonable request. All data needed are included in the paper.
